# Dynamic Cellular Cartography: Mapping the Local Determinants of Oligodendrocyte Transcription Factor 2 (OLIG2) Function in Live Cells Using Massively Parallel Fluorescence Correlation Spectroscopy Integrated with Fluorescence Lifetime Imaging Microscopy (mpFCS/FLIM)

**DOI:** 10.1021/acs.analchem.1c02144

**Published:** 2021-08-24

**Authors:** Sho Oasa, Aleksandar J. Krmpot, Stanko N. Nikolić, Andrew H. A. Clayton, Igor F. Tsigelny, Jean-Pierre Changeux, Lars Terenius, Rudolf Rigler, Vladana Vukojević

**Affiliations:** † Department of Clinical Neuroscience (CNS), Center for Molecular Medicine (CMM), Karolinska Institutet, 17176 Stockholm, Sweden; ‡ Institute of Physics Belgrade, University of Belgrade, 11080 Belgrade, Serbia; § Optical Sciences Centre, Department of Physics and Astronomy, School of Science, Swinburne University of Technology, Melbourne, Victoria 3122, Australia; ∥ Department of Neurosciences, University of California San Diego, La Jolla, California 92093-0819, United States; ⊥ Department of Neuroscience, Unité Neurobiologie Intégrative des Systèmes Cholinergiques, Institut Pasteur, F-75724 Paris 15, France; # Department of Medical Biochemistry and Biophysics (MBB), Karolinska Institutet, 17177 Stockholm, Sweden

## Abstract

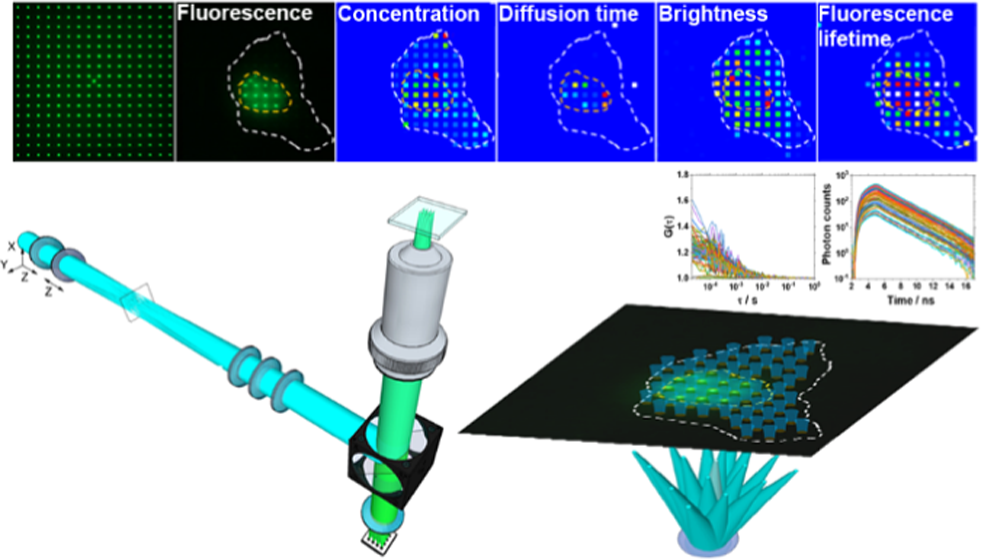

Compartmentalization and integration of molecular processes through diffusion are basic mechanisms through which cells perform biological functions. To characterize these mechanisms in live cells, quantitative and ultrasensitive analytical methods with high spatial and temporal resolution are needed. Here, we present quantitative scanning-free confocal microscopy with single-molecule sensitivity, high temporal resolution (∼10 μs/frame), and fluorescence lifetime imaging capacity, developed by integrating massively parallel fluorescence correlation spectroscopy with fluorescence lifetime imaging microscopy (mpFCS/FLIM); we validate the method, use it to map in live cell location-specific variations in the concentration, diffusion, homodimerization, DNA binding, and local environment of the oligodendrocyte transcription factor 2 fused with the enhanced Green Fluorescent Protein (OLIG2-eGFP), and characterize the effects of an allosteric inhibitor of OLIG2 dimerization on these determinants of OLIG2 function. In particular, we show that cytoplasmic OLIG2-eGFP is largely monomeric and freely diffusing, with the fraction of freely diffusing OLIG2-eGFP molecules being *f*_D,free_^cyt^ = (0.75 ± 0.10) and the diffusion time τ_D,free_^cyt^ = (0.5 ± 0.3) ms. In contrast, OLIG2-eGFP homodimers are abundant in the cell nucleus, constituting ∼25% of the nuclear pool, some *f*_D,bound_^nuc^ = (0.65 ± 0.10) of nuclear OLIG2-eGFP is bound to chromatin DNA, whereas freely moving OLIG2-eGFP molecules diffuse at the same rate as those in the cytoplasm, as evident from the lateral diffusion times τ_D,free_^nuc^ = τ_D,free_^cyt^ = (0.5 ± 0.3) ms. OLIG2-eGFP interactions with chromatin DNA, revealed through their influence on the apparent diffusion behavior of OLIG2-eGFP, τ_D,bound_^nuc^ (850 ± 500) ms, are characterized by an apparent dissociation constant *K*_d,app_^OLIG2-DNA^ = (45 ± 30) nM. The apparent dissociation constant of OLIG2-eGFP homodimers was estimated to be *K*_d,app_^(OLIG2-eGFP)2^ ≈ 560 nM. The allosteric inhibitor of OLIG2 dimerization, compound NSC 50467, neither affects OLIG2-eGFP properties in the cytoplasm nor does it alter the overall cytoplasmic environment. In contrast, it significantly impedes OLIG2-eGFP homodimerization in the cell nucleus, increasing five-fold the apparent dissociation constant, *K*_d,app,NSC50467_^(OLIG2-eGFP)2^ ≈ 3 μM, thus reducing homodimer levels to below 7% and effectively abolishing OLIG2-eGFP specific binding to chromatin DNA. The mpFCS/FLIM methodology has a myriad of applications in biomedical research and pharmaceutical industry. For example, it is indispensable for understanding how biological functions emerge through the dynamic integration of location-specific molecular processes and invaluable for drug development, as it allows us to quantitatively characterize the interactions of drugs with drug targets in live cells.

The intracellular environment is a complex and crowded, spatially heterogeneous medium the organization of which is bestowed and dynamically maintained through innumerable reaction-diffusion processes.^1,2^ While strong interactions (bond dissociation energies *D*_0_ > 20 kJ/mol) are important determinants of cellular physiology as they confer specificity and selectivity,^3^ it is well established that weak, nonspecific interactions (*D*_0_ < 20 kJ/mol), such as hydrogen bonding and interactions between permanent and transient dipoles, are equally important despite being so weak that they can be broken with energies that are within the range of thermal fluctuations. At the molecular level, weak interactions define macromolecular configuration and conformation, and hence, their function.^4^ At the cellular level, they are critical determinants of the overall organization of the cellular interior and significantly contribute to compartmentalization, i.e., the formation of distinct local environments (often called membrane-less organelles), where particular interactions between relevant biomolecules are enabled to efficiently proceed.^5−7^ The evolution of mechanisms that harness weak cooperative interactions was recently shown to render living organisms more capable of robustly undergoing evolutionary changes, and it appears that such mechanisms have been repeatedly positively selected during the evolution of increasingly complex organisms.^8^ The quest to deploy weak cooperative interactions is also of relevance for designing new drugs, in particular for the development of so-called allosteric drugs.^9−11^ Allosteric drugs exploit a fundamental mechanism, initially identified in multisubunit/multimeric proteins,^12−14^ which was later observed also in monomeric, intrinsically disordered proteins.^15^ They bind to a distant binding site, inducing rearrangements in the network of weak cooperative interactions that propagate across comparatively long distances, eventually rendering the active site more/less amenable for orthosteric ligand/drug binding.^16^ Efforts to develop allosteric drugs focus on understanding the function of natural molecules that act as allosteric modulators,^17^ rely on the use of computational approaches to identify allosteric binding sites that can be specifically targeted,^18,19^ and are inseparable from the advancement of experimental techniques to understand detailed molecular mechanisms that underlie allostery^20^ and to characterize the effects of prospective allosteric drug candidates.^21^ Experimental techniques designed to probe these processes in the cellular milieu need to be sensitive over a range of timescales (nanoseconds-to-seconds) and length scales (nanometers to microns).

Fluorescence correlation spectroscopy (FCS) and its dual-color variant fluorescence cross-correlation spectroscopy (FCCS) are the only presently available techniques that can nondestructively measure the concentration, diffusion, and binding of fluorescent/fluorescently labeled molecules in live cells with single-molecule sensitivity and high, sub-microsecond, temporal resolution.^22^ However, the same feature of FCS/FCCS that enables the ultimate, single-molecule sensitivity—the possibility to probe a minute observation volume element, thereby significantly reducing the background and improving the signal-to-background-ratio, confers also a serious limitation. Thus, conventional FCS/FCCS is of limited overview, i.e., measurements are restricted to a single-point location, probing in the cell a tiny volume of (0.2–2) × 10^–15^ l.^23−26^ To overcome this limitation, FCS was “amalgamated” with imaging-based methods, yielding new experimental techniques, such as temporal image correlation spectroscopy (TICS)^27^ and raster image correlation spectroscopy (RICS),^28,29^ which rely on raster scanning of the laser beam to illuminate a larger area; and single-plane illumination microscopy-based FCS (SPIM-FCS)^30−33^ and massively parallel FCS (mpFCS),^34−36^ which deploy different illumination strategies to cover a larger area. While these new techniques enable location-specific mapping of molecular concentration and diffusion in cells, they also entail some limitations. For example, the temporal and spatial resolution of TICS are inversely related and one is improved at the expense of the other—spatial resolution of TICS increases when the temporal resolution is in the millisecond range, due to long image plane acquisition time by raster scanning. This renders TICS either ill-suited for the study of fast processes or confers low spatial resolution.^27^ Similarly, RICS sacrifices spatial resolution to determine the diffusion and the number of molecules,^28,29,37^ as averaging over a relatively large number of pixels (>64) is needed to allow an accurate spatial correlation analysis. It also has significant problems when analyzing heterogeneous samples since the presence of bright speckles significantly deforms the autocorrelation curve. SPIM-FCS, which relies on the use of light-sheet illumination and a 2D camera to examine larger areas, can achieve high temporal resolution—recently reaching 6 μs for a reasonably short (≈100 s) measurement duration using the Swiss single-photon avalanche diode array (CHSPAD) camera.^32,38^ SPIM-FCS is, however, inherently hampered by the nonuniform thickness of the light sheet, which widens toward the edges, thus forming larger observation volume elements. Furthermore, scattering of the light sheet in heterogeneous environments and the presence of opaque compounds within the specimen alter the light-sheet intensity and can even completely block the incident light, which is recognized by the appearance of dark stripes in SPIM images. In SPIM-FCS, this translates to nonuniform illumination and hence a nonuniform signal-to-noise (SNR) ratio across the image. mpFCS relies on the spatial modulation of the incident laser beam by a diffractive optical element (DOE) to generate a large number of illumination spots, and a matching SPAD camera to detect in a confocal arrangement of the fluorescence intensity fluctuations from a large number (1024 in a 32 × 32 arrangement) of observation volume elements, providing single-molecule sensitivity and high spatial (∼250 nm) and temporal (21 μs) resolution.^34,35,39,40^ mpFCS was shown to be widely applicable, for the analysis of fast diffusion processes of eGFP-fused functional biomolecules in live cells^35^ and in live tissue *ex vivo*.^39^ The broad applicability of the mpFCS for functional fluorescence microscopy imaging (fFMI) was a motivation for us to go a step further and develop a new fFMI modality, mpFCS integrated with fluorescence lifetime imaging microscopy (mpFCS/FLIM). The fluorescence lifetime of a fluorophore or a fluorescently labeled macromolecule provides information on the environment local to the fluorophore (e.g., refractive index, polarity, pH, PO_2_, Ca^2+^). It can provide complementary insights into nanoscale (1–10 nm) macromolecular interactions or conformations via Förster resonance energy transfer (FRET) and dynamic quenching on the nanosecond timescale.

Here, we present an integrated massively parallel FCS and FLIM system (mpFCS/FLIM) on the same microscope frame. This enables massively parallel measurements to quantitatively characterize the location-specific concentration, mobility and interactions (via FCS), and local properties of the immediate surrounding of biomolecules (via fluorescence lifetime). We demonstrate the capabilities of mpFCS/FLIM for quantitative live cell biochemistry and cellular pharmacology by characterizing the effect of test compound NSC 50467 on oligodendrocyte transcriptional factor 2 (OLIG2) dimerization. OLIG2, a basic helix–loop–helix transcription factor in the central nervous system, plays an important role in neuronal cell differentiation during development,^41^ adult neurogenesis,^42^ and glioblastoma development.^43^ Substances that target OLIG2 are attractive candidates for the development of therapeutic agents for glioblastoma.^44^ However, identification of such molecules is not trivial due to the large and complex surface through which OLIG2 interacts with itself and other partners, which is uncharacteristic and with no hydrophobic pockets.^18,19^ The NSC 50467 compound was identified *in silico* using the so-called “combined pharmacophore approach” and was predicted to act as an allosteric inhibitor of OLIG2 homodimerization^45−47^ thus impeding OLIG2 homodimer binding to the enhancer box (E-box), which is the canonical bHLH transcription factor binding site.^45−47^

## Experimental Section

### Optical Setup for Massively Parallel Fluorescence Correlation Spectroscopy Integrated with Fluorescence Lifetime Imaging Microscopy (mpFCS/FLIM)

**Figure 1:**
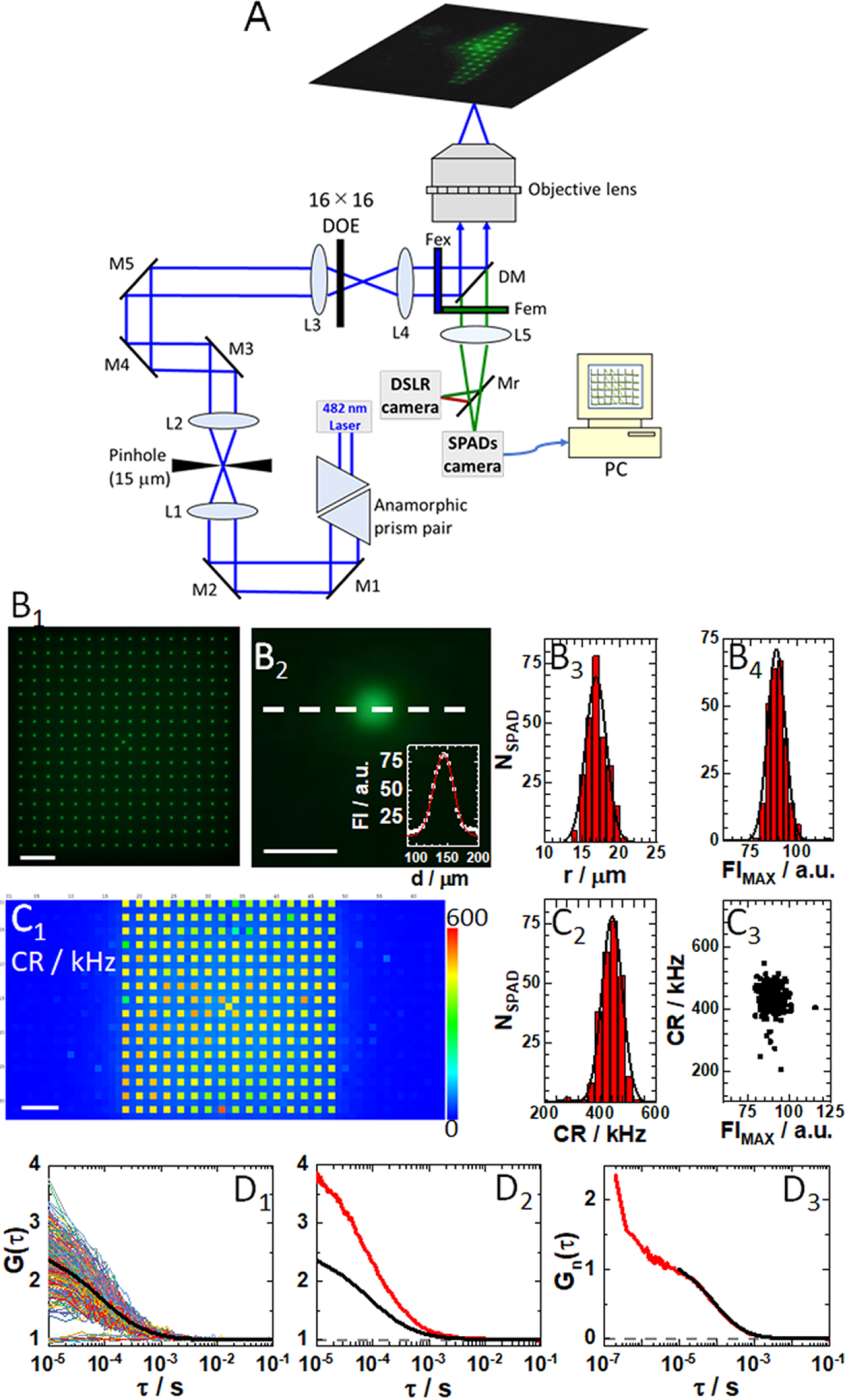
Optical setup for mpFCS/FLIM. (A) Schematic drawing of the mpFCS/FLIM optical setup. The 482 nm laser beam with elliptical cross-section is transformed into a circular beam using an anamorphic prism pair and expanded using a Kepler telescope setup (L1 and L2) with a pinhole in its focus. The expanded circular laser beam is focused by the focusing lens (L3) mounted on an *xyz* translation stage, which is positioned in front of the diffractive optical element (DOE) that can be translated along and rotated around the *z*-axis. The illumination matrix consisting of 16 × 16 (256) spots, which are generated in the image plane of the back port of the microscope, is imaged by the microscope relay optics (L4) and the objective lens to the object plane. Fluorescence is detected by a single-photon avalanche diode (SPAD) camera that can be translated along the *z*-axis and tilted at two angles (pitch and yaw) or a digital single-lens reflex (DSLR) camera. (B_1_) Image of the illumination matrix visualized by the DSLR camera using a thin fluorescence layer as a specimen. (B_2_) Enlarged image of a single illumination spot shown in (B_1_). Inset: Fluorescence intensity (FI) distribution through the center of the spot (white dashed line) and the best-fit Gaussian curve (red solid line). Spot roundness, assessed by measuring the spot radius in different directions: horizontal (0°; white dashed line), 45, 90, and 135°, showed that the ratio of spot radius over the spot radius at 0° was 1.00, 1.02, 0.96, and 1.04, respectively. (B_3_) Histogram of spot radii for all 256 spots in the confocal image of the illumination matrix is shown in (B_1_). The average spot radius, *r*_spot_ = (17 ± 2) μm, was determined from a half of the full width at half-maximum (FWHM) of the best-fit Gaussian curve. (B_4_) Histogram of peak fluorescence intensity for all spots in the confocal image of the illumination matrix is shown in (B_1_). The average peak fluorescence intensity, FI_MAX_ = (90 ± 5) au. (C_1_) Scanning-free confocal image of the same specimen as in (B_1_) acquired using the SPC^3^ SPAD camera. Here, each SPAD in addition to being a photodetector also acts as a 30 nm pinhole. Of note, every other SPAD in the centrally positioned 32 × 32 SPADs of the 64 × 32 SPC^3^ SPAD camera was used. Unilluminated SPADs (dark blue), on the sides and in-between the illuminated ones (yellow–red ones), are clearly distinguishable by fluorescence intensity. (C_2_) Histogram of fluorescence intensity, i.e., photon count rates (CR) measured in all illuminated SPADs shown in (C_1_). The average fluorescence intensity was determined, CR = (440 ± 35) kHz. (C_3_) Scatter plot showing spot peak intensity measured using the SPC^3^ SPAD camera (C_1_) as compared to the spot intensity measured using the DSLR camera (B_1_). While a unimodal distribution is observed, six SPADs with disparate values were identified. (D_1_) 256 single-SPAD autocorrelation curves (ACCs) recorded in an aqueous buffer solution of eGFP, *c*_eGFP_ = 4 nM, with the corresponding average ACC (black). (D_2_) ACCs acquired in the same solution as in D_1_ by mpFCS (black; same as in D_1_) and spFCS ACC (red). The dashed gray line shows *G*(τ) = 1. (D_3_) ACCs shown in D_2_ normalized to the same amplitude, *G*(10 μs) = 1 at τ = 10 μs, acquired using the spFCS (red) and the mpFCS (black) systems. The dashed gray line shows *G_n_*(τ) = 0. In all images, scale bar is 10 μm.

The optical design of the mpFCS/FLIM system and important features are shown in Figure 1A–C_3_. Information about optical alignment, calibration, data acquisition, analysis, image rendering, and fitting of temporal autocorrelation curves (ACCs) using eq S1 is provided in the Supporting Information (Section S1, Figures S1–S5).

### Software for mpFCS/FLIM

mpFCS/FLIM data acquisition, analysis, and graphical presentation were carried out using our own software, into which the Micro Photon Device (MPD) software for running the 2D SPAD array was integrated. The software was written in Embaracadero C++ Builder 10.2 (Embarcadero Technologies). Detailed information about data acquisition, analysis, and image rendering are given for mpFCS in Section S1b and for FLIM in Section S1c. Phasor plot analysis is presented in Section S1d.

### Cell Culture and Transfection

Procedures for cell culturing and transfection for mpFCS/FLIM measurements (Section S2a), pharmacological treatment of cells (Section S2b), and cell culture for FRET-FLIM measurements (Section S2c) can be found in the indicated sections in the Supporting Information.

### Dissociation Constant Assessment

Procedures for calculating the apparent dissociation constants of OLIG2-eGFP dimers (Section S3) and Olig2-eGFP–DNA complexes (Section S4) can be found in the indicated sections in the Supporting Information.

### Standard Solutions for mpFCS/FLIM Calibration

Relevant information about standard solutions used for mpFCS/FLIM system calibration can be found in Section S5.

### Statistical Analysis

All values are presented as mean ± standard deviation (SD). Two-tailed Student’s *t*-test was used to compare two groups. The correlation analyses were reported using the probability value (*p*-value). Differences between two groups were considered to be significant when *p* < 0.05. Pearson’s sample correlation coefficient *r* was used to assess the strength of a linear association between two variables. Statistical analysis was performed using the Origin 2018 program for interactive scientific graphing and data analysis and/or Excel. During data analysis, data from a few pixels (<5%) were disregarded due to the extremely high background in these SPADs. The results were replicated in three independent experiments, starting from cell transfection, culturing, treatment, and measurement. Similar trends were observed in all three experiments. Figures show representative data acquired in a single cell.

## Results

### Validation of mpFCS/FLIM System Performance for FCS

The sensitivity and temporal resolution of the mpFCS/FLIM system are unprecedented, enabling us to perform measurements in a buffered aqueous solution of the enhanced Green Fluorescent Protein (eGFP; Figure 1D_1_–D_3_). Of note, the amplitude of the average ACC acquired by mpFCS is half the amplitude of the ACC acquired using conventional single-point FCS (spFCS), largely due to a higher background in the mpFCS system than in the spFCS system (Figure 1D_2_). In contrast, normalized autocorrelation curves nicely overlap (Figure 1D_3_), revealing that the observation volume elements (OVEs) in the mpFCS and the spFCS systems are of similar size. We also show that the ACC can be fitted with the acceptable signal to noise using eq S1, α = 1, *i* = 1, *T* = 0 (Figure S4A,B) and that the axial ratio is not diverging (*s* = ω_*z*_/ω_*xy*_ = 4.6), which indicates that the assumption of a 3D-ellipsoidal Gaussian OVE is applicable. Finally, we show by *z*-stack imaging that the fluorescence intensity profile in the axial direction is Gaussian with a half width at half-maximum, HWHM = (1.15 ± 0.09) μm (Figure S4C).

### Validation of mpFCS/FLIM System Performance for FLIM

**Figure 2:**
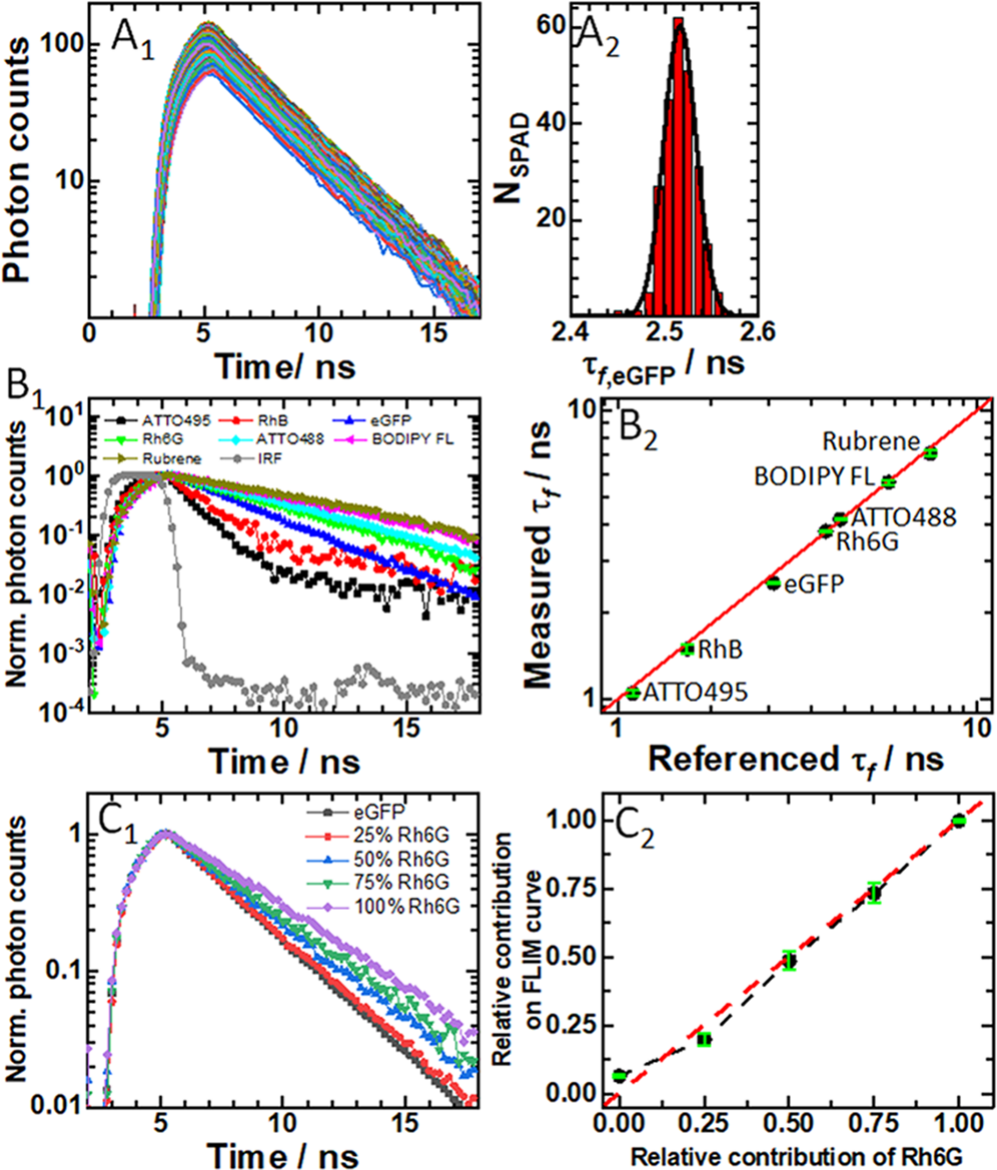
Fluorescence lifetime imaging microscopy (FLIM) using the integrated mpFCS/FLIM system. (A_1_) 256 simultaneously recorded eGFP fluorescence decay curves in aqueous phosphate buffer. (A_2_) Corresponding histogram of fluorescence lifetimes obtained using a one-component exponential decay model to fit the fluorescence decay curves. From a best-fit Gaussian curve, the fluorescence lifetime was determined, τ_*f*,eGFP_ = (2.50 ± 0.02) ns. (B_1_) Fluorescence decay curves recorded in aqueous solutions of different fluorescent dyes: ATTO495 (black), Rhodamine B (RhB; red), eGFP (blue), Rhodamine 6G (Rh6G; green), ATTO488 (cyan), BODIPY FL (magenta), Rubrene (dark yellow), and the Instrumental Response Function (IRF; gray), all acquired using the same SPAD in the SPC3 camera. (B_2_) Comparison of fluorescence lifetimes measured using the mpFCS/FLIM system with literature values. Pearson’s correlation indicated that there was a significant positive association between the measured and literature values (*r*(7) = 0.999, *p* < 0.001). The red line indicates perfect agreement. (C_1_) Normalized fluorescence decay curves for Rh6G, eGFP, and their mixtures made so that a specified number of photons originates from Rh6G, e.g., 50% Rh6G indicates that 50% of photons are from Rh6G: eGFP (0% Rh6G; dark gray), 25% Rh6G (red), 50% Rh6G (blue), 75% Rh6G (green), and 100% Rh6G (violet). (C_2_) Comparison of the relative contribution of Rh6G, as determined from fluorescence lifetime measurements using a two-component exponential decay fitting model with fixed fluorescence lifetimes: τ_f,eGFP_ = 2.5 ns and τ_f,Rh6G_ = 3.8 ns (black dots), with its actual concentration in a two-component mixture. Pearson’s correlation indicated that there was a significant positive association between the measured τ_f_ and values found in the literature (*r*(5) = 0.995, *p* < 0.01).

To characterize the performance of the mpFCS/FLIM system for fluorescence lifetime (τ_f_) measurements, the instrument response function (IRF) was measured and single-exponential decay fitting of FLIM curves was compared to convolution fitting with the IRF (Figure S6); effects of the gate width and the step size between gates on τ_f_ were examined (Figure S7); the precision with which τ_f_ of pure species can be determined was assessed using solutions of molecules with known fluorescence lifetimes (Figures 2 and S8); and the ability of our system to resolve two lifetimes using measurements at a single frequency was evaluated using a series of two-component solutions with different relative contributions of the two component (Figures 2 and S9). The most important results are summarized in Figure 2.

Briefly, Figure 2A_1_ shows 256 simultaneously recorded fluorescence decay curves in a phosphate buffer solution of eGFP. Analysis using the single-exponential decay model (eq S2) yielded a histogram of fluorescence lifetimes from which eGFP fluorescence lifetime was determined, τ_f,eGFP_ = (2.5 ± 0.02) ns (Figure 2A_2_). This value agrees well (i.e., to within 10%) with the values obtained in other laboratories.^48−50^

Using a 2.0 ns gate width and a 0.2 ns gate step time, τ_f_ was measured for several standards in solution, covering a τ_f_ range from 1 to 10 ns (Figure 2B_1_,B_2_). The agreement between expected and measured fluorescence lifetimes, which can be gleaned from Figure 2B_2_, is excellent (*r* = 0.999, *p* < 0.001).

Given that τ_f_ can be considered a “molecular fingerprint,” allowing detection and discrimination between multiple species that emit fluorescence over the same spectral window, we tested the capability of our instrument to distinguish fluorophores that emit in the same spectral region and have discernible lifetimes, Rhodamine 6G (Rh6G), τ_f,Rh6G_ = (3.80 ± 0.04) ns, and eGFP, τ_f,eGFP_ = (2.50 ± 0.02) ns. To this aim, we mixed Rh6G and eGFP solutions at different proportions (Figure 2C_1_,C_2_). As expected, the total τ_f_ increased as the proportion of the species with the longer τ_f_ (here Rh6G) was increased (Figure 2C_1_). A fit of the data to an exponential decay function by two processes (eq S3; with τ_f_ for eGFP and Rh6G fixed and amplitudes floated) yielded relative amplitudes that matched well the calculated relative contribution of the components in the mixture (Figure 2C_2_).

Since attempts to fit the data with a two-component-exponential decay model with free-floating τ_f_ and their relative contributions did not lead to extraction of the correct component lifetimes and their relative amounts (Figure S9A_1_,A_2_), phasor analysis^51−53^ was used to analyze the simultaneously acquired fluorescence decay curves, assuming that two lifetime components were common to all of the curves. By deploying phasor analysis, which uses the Fourier transform to decompose experimentally measured fluorescence decay curves into complex-valued functions of the modulus (*m*) and the phase angle (θ_tot_) (eqs S4–S21), global analysis of a two-component system is reduced to algebraic calculations in the phasor space (Figure S9B_3_). Following calibration experiments (Figure S8), we computed by phasor analysis τ_f_ and components’ fractions with dramatically improved accuracy and precision (Figure S9B_1_,B_2_).

### Spatial Mapping of Fluorescence Lifetime in a Fixed Specimen

**Figure 3:**
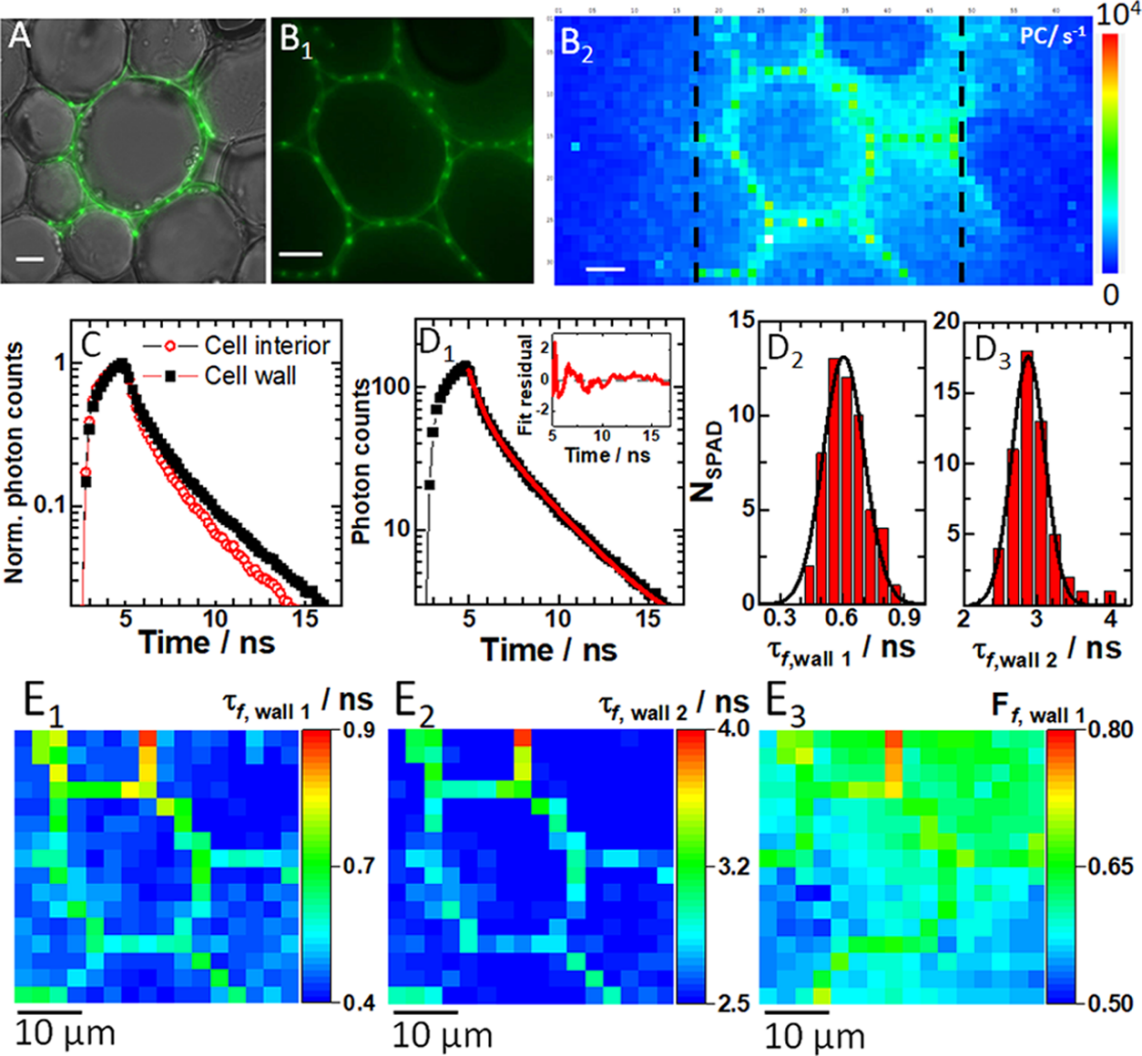
Spatial distribution of fluorescence lifetime in a fixed section of the rhizome of lily of the valley (*C. majalis*). (A) Fluorescence image of a spot-wise, 16 × 16, illuminated cell (green) overlaid on a wide-field transmission image (gray) of a region in the central parenchyma recorded using the DSLR camera. (B_1_) Zoomed fluorescence image of a spot-wise illuminated cell in the central parenchyma recorded using the DSLR camera. (B_2_) Fluorescence image of the same cell as in (B_1_) acquired using the SPC^3^ SPAD camera. Fluorescence intensity is given in photon counts (PC), exposure time 46 ms. (C) Fluorescence decay curves recorded in individual SPADs at distinct intracellular locations: cell wall (black squares) and inside the cell (red circles). All fluorescence decay curves are shown in Figure S10. (D_1_) A fluorescence decay curve recorded in an individual SPAD at the cell wall (black squares) fitted using a two-component exponential decay model (eq S3, red line). Inset: Corresponding residuals. (D_2_) Histogram of the short fluorescence lifetime component in the plasma membrane and the best-fit Gaussian curve yield τ_f,wall1_ = (0.6 ± 0.1) ns. (D_3_) Histogram of the long fluorescence lifetime component in the plasma membrane and the best-fit Gaussian curve yield τ_f,wall2_ = (2.9 ± 0.2) ns. (E_1–3_) FLIM images of the cell in (B_2_) rendered visible by mapping the: short (E_1_) and long (E_2_) fluorescence lifetime component and the relative contribution of the short component (E_3_). In all images, the scale bar is 10 μm.

To demonstrate spatial mapping of τ_*f*_, a fixed plant specimen, the acridine orange stained section through the rhizome of the lily of the valley (*Convallaria majalis*) was used (Figure 3).

Fluorescence images acquired using a spot-wise, 16×16, illumination and the DSLR camera (Figure 3A,B_1_) show cells in the central parenchyma made visible owing to the fluorescence signal from the cell wall. The fluorescence image of the same cell as in Figure 3B_1_ acquired using the SPC^3^ SPAD camera is shown in Figure 3B_2_. Fluorescence decay curves simultaneously recorded in 256 individual SPADs (Figure S10), exemplified in Figure 3C, when fitted using a two-component exponential decay model (Figure 3D_1_), yielded a short, τ_f,wall1_ = (0.6 ± 0.1) ns (Figure 3D_2_) and a long fluorescence lifetime component, τ_f,wall2_ = (2.9 ± 0.2) ns (Figure 3D_3_). Importantly, the thus determined τ_f_ provided significant image contrast (Figure 3E_1_,E_2_), and even a “ratiometric” image could be obtained revealing the relative contribution of the component with the short fluorescence lifetime (Figure 3E_3_).

### Spatial Mapping of Concentration, Diffusion, and Fluorescence Lifetime in Live Cells

**Figure 4:**
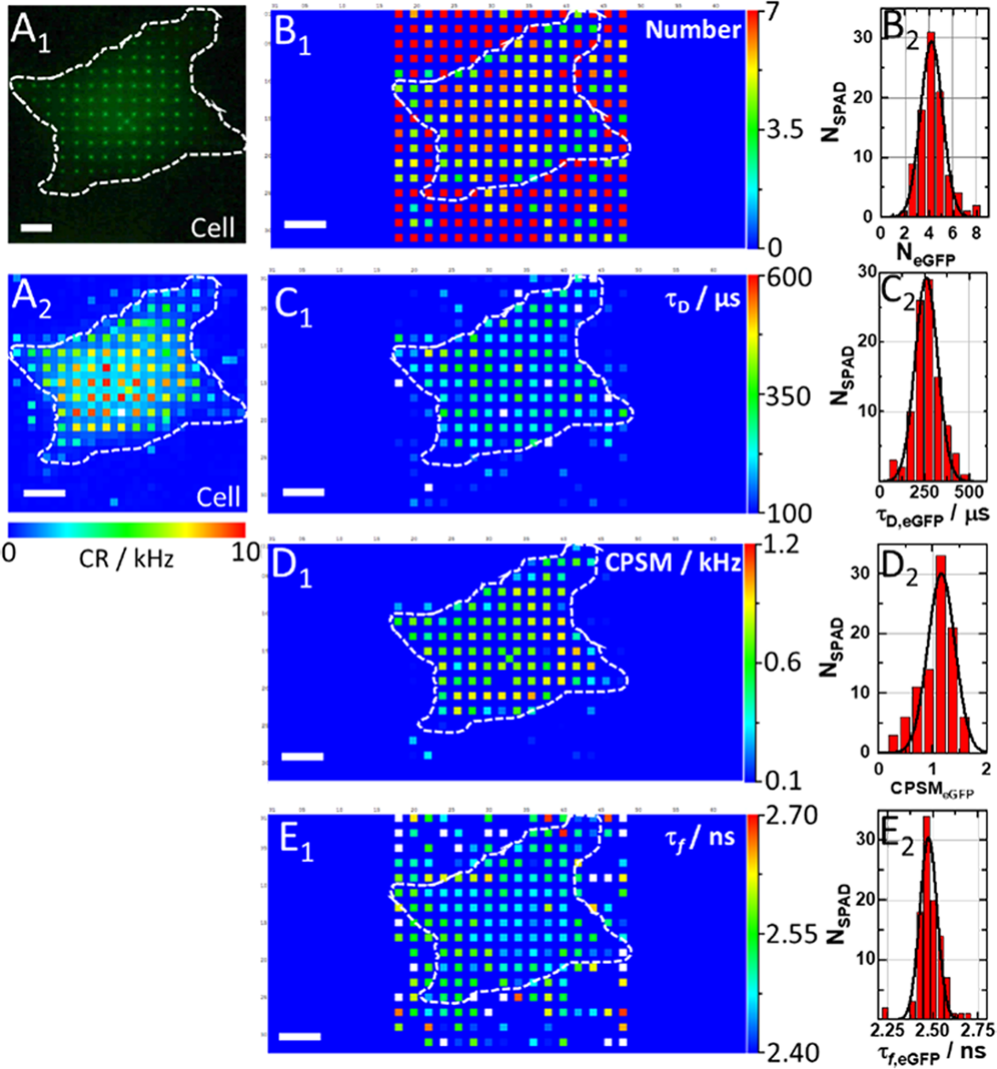
Spatial map of eGFP concentration, diffusion, brightness, and fluorescence lifetime in a live HEK cell. (A_1_) Fluorescence image of an eGFP-expressing HEK cell acquired using a spot-wise, 16 × 16, illumination and a DSLR camera. The hand-drawn dashed line highlights the cell border visualized by transmission light imaging. (A_2_) Count rate map. Corresponding ACCs and FLIM curves are shown in Figure S12. (B_1_) Spatial map of the average *N*_eGFP_ in the OVE. Of note, the apparently high average number of molecules in the cell surrounding is an artifact of the near-zero amplitude of the ACCs in the cell culture medium (see Figure S12A_2_). (B_2_) Histogram corresponding to B_1_. The best-fit Gaussian curve yields *N*_eGFP_ = (4.22 ± 0.92), corresponding to *c*_eGFP_ ≈ 20 nM. (C_1_) Spatial map of τ_D,eGFP_. (C_2_) Histogram corresponding to (C_1_) yields the average eGFP diffusion time, τ_D,eGFP_ = (260 ± 60) μs. (D_1_) Spatial map of eGFP brightness as reflected by counts per second per molecule (CPSM). (D_2_) Histogram corresponding to (D_1_) yields average CPSM_eGFP_ = (1.0 ± 0.3) kHz. (E_1_) Spatial map of eGFP fluorescence lifetimes. (E_2_) Histogram corresponding to (E_1_) yields the average eGFP fluorescence lifetime, τ_f,eGFP_ = (2.50 ± 0.05) ns.

To demonstrate spatial mapping of the concentration, diffusion, and lifetime in live cells (Figure S11), we first performed measurements on fluorescent proteins, eGFP (Figures 4 and S12) or eGFP tetramer (eGFP_tet_; Figures S13 and S14), as nonreactive molecular probes.

Our data show that for similar eGFP concentrations in the cell, *c*_eGFP_ ≈ 20 nM, and in the aqueous buffer solution, *c*_eGFP_ ≈ 4 nM, the mean diffusion time of eGFP was about 2.5 times longer in the cell than in the aqueous buffer, τ_D,eGFP,cell_ = (260 ± 60) μs vs τ_D,eGFP,buffer_ = (110 ± 10) μs, consistent with previous studies,^54^ whereas the molecular brightness and fluorescence lifetimes were similar CPSM_eGFP,cell_ = (1.0 ± 0.3) kHz and CPSM_eGFP,buffer_ = (1.0 ± 0.2) kHz, τ_f,eGFP,cell_ = (2.50 ± 0.05) ns and τ_f,eGFP,buffer_ = (2.50 ± 0.02) ns. However, the relative standard deviations (RSD) of all measured variables were higher in the living cell (Figure 4B_2_,C_2_,D_2_,E_2_) than those in the homogenous solution (Figure S3B–E), indicating that the cell environment presents a spatial variation in local concentration, local diffusion processes (Figure S15), and local excited-state decay (environment). Correlation maps (Figure S16) showed that no correlation was observed between the concentration (number of eGFP), molecular brightness, and lifetime, ruling out any spatially dependent concentration quenching in the fluorescence lifetime and absence of diffusion-influenced lifetime quenching. Taken together, these results are largely consistent with the view of eGFP being a biochemically inert, monomeric protein, able to roam largely unimpeded inside the cellular milieu. The broadened distribution functions observed here (relative to homogenous aqueous buffer) reveal that the cellular interior is not uniform and that eGFP is not totally confined to the cytosol but is also found in cytoplasmic organelles.

In contrast to eGFP, which can access the entire cell, a fluorescence image of a HEK cell-expressing eGFP_tet_ reveals distinctive fluorescence intensities in the cytoplasm and the cell nucleus (Figures S11B and S13A). Furthermore, the large RSD of the diffusion time for eGFP_tet_ in the cytoplasm is of particular note, as it is ten-fold larger than the corresponding value for the monomeric eGFP in the cytoplasm. Because the eGFP_tet_ is 4 times larger than eGFP (4 nm long axis dimension), this suggests that obstacles in the size range of 10 nm or more in the cellular environment affect eGFP_tet_ dynamics, as revealed using the anomalous diffusion model (eq S1, α ≠ 1)^55−57^ to fit the experimentally derived ACCs and determine the anomalous diffusion exponent (α; Figure S15B). Furthermore, and in contrast to the diffusion time, the fluorescence lifetime was homogeneous in cells expressing eGFP_tet_ (Figure S13E_1_). FLIM curves in the nucleus showed lower photon counts but revealed similar decay rates (Figure S14C_1–3_). The histogram of fluorescence lifetime quantified τ_f,eGFPtet_ = (2.4 ± 0.05) ns in the cytoplasm and similarly in the nucleus (Figure S13E_2_).

### Spatial Mapping of Transcription Factor OLIG2-eGFP in Live Cells Before and After Treatment with Compound NSC 50467: an Allosteric Inhibitor of OLIG2 Dimerization

To demonstrate spatial mapping of the concentration, diffusion, and lifetime of interacting molecules in live cells, intracellular localization and dynamics of OLIG2 was characterized (Figures 5, S11C,D, and S17–S25).

**Figure 5:**
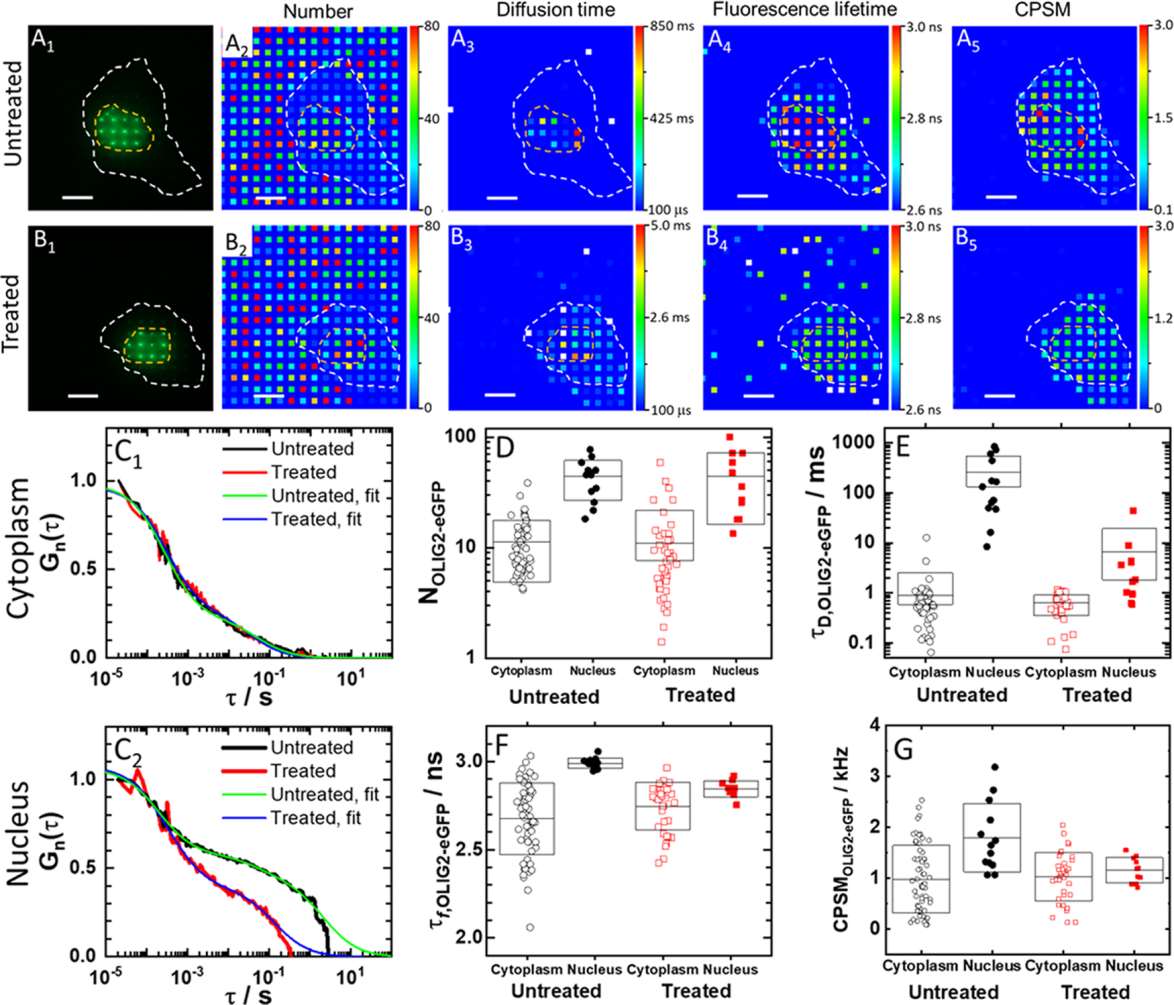
Spatial map of OLIG2-eGFP concentration, diffusion, brightness, and fluorescence lifetime in a live HEK cell before and after treatment with NSC 50467. (A_1_, B_1_) Fluorescence images of an untreated (A_1_) and a treated (B_1_) HEK cell-expressing OLIG2-eGFP, acquired using a spot-wise, 16 × 16, illumination and a DSLR camera. The hand-drawn dashed lines that highlight the cell border (white) and the cell nucleus (orange) were visualized by transmission light microscopy. Corresponding fluorescence intensity fluctuation time series and ACCs are shown in Figure S18. (A_2_, B_2_) Spatial map of the average number of OLIG2-eGFP molecules in an OVE, recorded in an untreated (A_2_) and a treated (B_2_) cell. (A_3_, B_3_) Spatial map of OLIG2-eGFP diffusion times recorded in an untreated (A_3_) and a treated (B_3_) cell. (A_4_, B_4_) Spatial map of fluorescence lifetimes recorded in an untreated (A_4_) and a treated (B_4_) cell. Corresponding FLIM curves are shown in Figure S18. (A_5_, B_5_) Spatial map of OLIG2-eGFP brightness (CPSM) recorded in an untreated (A_5_) and a treated (B_5_) cell. (C_1_, C_2_) Single-pixel ACCs normalized to the same amplitude, *G*(20 μs) = 1 at τ = 20 μs, recorded in the same pixel in the cytoplasm (C_1_) and the same pixel in the cell nucleus (C_2_) before (black) and after (red) treatment. Two-component 3D free diffusion model fitting to the ACCs recorded in the cell nucleus and the cytoplasm in the untreated (green) and the treated (blue) cell. (D–G) Effect of treatment on the number of molecules (D), diffusion time (E), average fluorescence lifetime (F), and average molecular brightness (G).

OLIG2 is known to bind as a homodimer to the enhancer box (E-box), the canonical bHLH transcription factor binding site.^45−47^ It is predominantly localized in the cell nucleus (Figures 5A_1_ and S11C), but is known to shuttle between the nucleus and the cytoplasm (Figure S11D), with the actual localization pattern emerging from a dynamic equilibrium that is predominantly governed by the nuclear export signal.^58^ Spatial mapping of the number of OLIG2-eGFP in untreated cells revealed that the concentration of OLIG2-eGFP in the cell nucleus is higher than in the cytoplasm (Figure 5A_2_); the diffusion time, determined from the full width of the ACC at half maximum, is significantly longer in the cell nucleus than in the cytoplasm, τ_D_^nuc^ = (250 ± 300) ms vs τ_D_^cyt^ = (0.9 ± 1.5) ms (Figure 5A_3_,E), and the fluorescence lifetime map revealed a significantly longer lifetime states in the cell nucleus, fluorescence lifetime, τ_f,OLIG2-eGFP_^nuc^ = (3.0 ± 0.3) ns vs τ_f,OLIG2-eGFP_^cyt^ = (2.7 ± 0.2) ns (Figure 5F), reflecting differences in the local environment surrounding the eGFP probe of OLIG2-eGFP in these cellular locations (Figure 5A_4_). Given the unexpectedly large experimental errors for diffusion times, we further examined ACCs. This analysis revealed two characteristic decay times in both, the cytoplasm (Figures 5C_1_ and S18B_1_,C_1_) and the cell nucleus (Figures 5C_2_ and S18B_2_,C_2_), with the fast-decaying components being, within the experimental error, indistinguishable between these compartments, τ_D,free_^cyt^ = τ_D,free_^nuc^ = (0.5 ± 0.3) ms, while the relative amplitude and the diffusion time of the second component were larger and much longer in the cell nucleus than in the cytoplasm, *f*_D,bound_^nuc^ = (0.65 ± 0.10) vs *f*_D,bound_^cyt^ = (0.25 ± 0.10) and τ_D,bound_^nuc^ = (850 ± 500) ms vs τ_D,bound_^cyt^ = (60 ± 30) ms, respectively. (Of note, fluorescence intensity time series (Figure S18A_1_,A_2_) show that the signal intensity is unchanged over time and is not distorted by photobleaching. Rather, the ACCs recorded in the cell nucleus do not settle at 1 because the decay time of the second component is comparable to the signal acquisition time length (20 s).) Finally, OLIG2-eGFP molecular brightness in the cytoplasm, CPSM_OLIG2-eGFP_^cyt^ = (1.0 ± 0.7) kHz (Figure 5G), was within the experimental error indistinguishable from that of eGFP in live cells, CPSM_eGFP_ = (1.0 ± 0.3) kHz, measured under the same conditions, suggesting that OLIG2-eGFP is monomeric in the cytoplasm. In the nucleus, average molecular brightness is higher, CPSM_OLIG2-eGFP_^nuc^ = (1.4 ± 0.7) kHz (Figure 5G and Table S1), suggesting that a dynamic equilibrium between OLIG2-eGFP monomers and dimers exists.

Treatment with the allosteric inhibitor of OLIG2 dimerization did neither change the concentration, nor the diffusion time, nor the fluorescence lifetime, and nor the molecular brightness of OLIG2-eGFP residing in the cytoplasm; *p*> 0.05 for all measurements (Figures 5C_1_, D–G, S17A_1–3_ and S19A_1–4_). However, it significantly perturbed the motions and the local environment of OLIG2-eGFP in the cell nucleus, causing, on the average, a decrease in the diffusion time by 4 times (from 850 to 200 ms; Figures 5C_2_,E and S19B_2_, *p* = 5 × 10^–3^), and reduced the fluorescence lifetime (Figure 5F, *p* = 1.5 × 10^–8^) and the molecular brightness (Figures 5G, S17B_3_ and S19B_4_, *p* = 7 × 10^–3^), while leaving the overall OLIG2-eGFP concentration unchanged, as reflected by the number of OLIG2-eGFP molecules (Figures 5D and S17B_1_, *p* > 0.05). Moreover, the positive correlation between local OLIG2-eGFP molecular brightness and the local diffusion, which was strong in the cell nuclei of untreated cells, was significantly reduced (Figure S19B_2-2_,_3-2_).

Finally, mpFCS measurements enabled us to assess the value of the apparent dissociation constants for OLIG2-eGFP binding to chromatin DNA before, *K*_d,app_^OLIG2-DNA^ = (45 ± 30) nM, and after treatment, *K*_d,NSC50467_^OLIG2-DNA^ = (130 ± 40) nM (Figure S19C_1–3_). Also, mpFCS measurement of OLIG2-eGFP concentration and molecular brightness revealed that in untreated cells about 25% of OLIG2-eGFP molecules are homodimers and that treatment with NSC 50467 effectively reduced OLIG2-eGFP homodimer levels to below 7% (Table S1). This, in turn, enabled us also to infer apparent OLIG2-eGFP homodimer dissociation constants in untreated cells *K*_d,app_^(OLIG2–eGFP)2^ ≈ 560 nM, which upon treatment becomes *K*_d,app,NSC50467_^(OLIG2-eGFP)2^ ≈ 3 μM.

Taken together, the mpFCS data indicate that treatment with the allosteric modulator NSC 50467 does not significantly alter OLIG2-eGFP properties in the cytoplasm, whereas in the cell nucleus OLIG2-eGFP dimers are not efficiently formed in the presence of NSC 50467 and OLIG2-eGFP binding to the chromatin DNA is significantly abolished.

We then used Förster resonance energy transfer (FRET) via FLIM (FRET-FLIM), to further characterize NSC 50467 effects on OLIG2-eGFP dimer formation. To this aim, cells expressing OLIG2-eGFP, with eGFP acting as a FRET donor, and dark yellow fluorescent protein ShadowY tagged OLIG2 molecules (OLIG2-ShY), with ShY acting as FRET acceptor, were used. For a positive FRET control, a tandem dimer of eGFP and ShadowY (eGFP-ShY) was transfected into cells. As expected, robust FRET was observed with the positive FRET control probe, with a FRET efficiency of 55% (as determined by phasor analysis of the FLIM data, Figure S20). Phasor plots recorded in cells expressing OLIG2-eGFP and OLIG2-ShadowY showed evidence of emission from a mixture of FRET and non-FRET states including the FRET contribution from the OLIG2 dimer (Figure S21A). In the context of a FRET/non-FRET state model (involving donor, acceptor, and FRET states), our analysis delivered an amplitude fraction of FRET to be (0.3 ± 0.1) in the absence of allosteric inhibitor, which decreased to (0.07 ± 0.06) upon treatment with the inhibitory compound, also observable at other cells (Figure S21B,E,F). As expected, the decrease in the FRET fraction was accompanied by an increase in the contribution of non-FRET states. This data provides evidence for the efficient inhibition of OLIG2 dimer formation by the inhibitory compound. Since the RSD of the amplitude of the FRET fraction of OLIG2 without compound is larger than that of the tandem dimer of fluorescent proteins (eGFP-ShY), we can conclude that OLIG2 dimerization in the nucleus was in addition to OLIG2 dimerization inhibition also affected by the nuclear environment (e.g., genome DNA structure).

## Discussion

In this work, we present two important achievements, the development of a new functional fluorescence microscopy imaging (fFMI) modality attained by integrating massively parallel fluorescence correlation spectroscopy with fluorescence lifetime imaging microscopy (mpFCS/FLIM) and demonstrate its use to characterize the action of a compound with potential therapeutic effects that target OLIG2.

Our instrument is a quantitative scanning-free confocal fluorescence microscope with single-molecule sensitivity; it has similar confocal volume elements with single-point FCS and 10 μs/frame temporal resolution and can map fluorescence lifetimes from 1 to 10 ns. The instrument builds on our previous work,^35^ but we have now improved to longer signal acquisition duration, ∼10 s from previous 2.7 s, with a higher temporal resolution, ∼10 μs/frame from previous ∼21 μs/frame, toward tracking faster dynamic processes. In addition, the SNR was dramatically improved. In particular, the number of particles ratio against spFCS reduced 10 times, from 50 to 5 for fluorospheres (*d* = 100 nm). Also, single-pixel autocorrelation curves in eGFP and QD525 in water agree to within 10% with spFCS. Importantly, the system integrated with FLIM enabled us to perform mpFCS and FLIM at the same position in the cell. This is a significant improvement compared to current practice, where considerable time lags are introduced when moving the specimen from one microscope to the other. At the same time, the time needed for finding the same cell after moving the specimen from one microscope to the other is entirely abolished. Our dedicated software provides mono- and two-component exponential decay fitting for all 256 SPADs nearly instantly, rendering a fluorescence lifetime image in a few seconds. Implementation of phasor analysis makes multicomponent analysis in FLIM easily achieved without the need to fit multicomponent exponential decay curves.

In comparison with other presently available 2D FCS instruments, such as FCS based on total internal reflection (TIR-FCS^59−62^) and single plane illumination microscopy (SPIM-FCS^30−33^), our approach is more versatile. The main limitation of TIR-FCS is its restriction to an investigation of processes at the basal plasma membrane. SPIM-FCS, on the other hand, enables us to visualize the inside of cells and perform measurements there, but it is hampered by an inhomogeneous illumination and is characterized by a relatively larger observation volume (∼1 × 10^–15^ l). Advantages of our approach are optical sectioning, homogenous illumination and detection, and small confocal volume elements (∼0.35 × 10^–15^ l), which is particularly important since larger observation volume elements average local differences in concentration, mobility, and intermediate surrounding of molecules in a live cell. Thus, the integrated mpFCS/FLIM system uniquely enables us to map with great precision the molecular numbers and mobility via mpFCS and characterize the local environment immediately surrounding fluorescent/fluorescently labeled molecules via FLIM. Instrument performance was stringently assessed in a series of validation experiments using well-characterized samples. Most notably, we have demonstrated that we could measure the concentration and diffusion of eGFP in a dilute aqueous solution (*c*_eGFP_ = 4 nM, Figure 1D_1_–D_3_) and showed that it is uniform (Figures S3 and S15). We have also shown that noninteracting molecules smaller than 5 nm, e.g., eGFP, diffuse without significant hindrance through the entire cell (Figures 4 and S15), while molecules/molecular complexes that are larger than 10 nm, such as eGFP_tet_, largely reside in the cytoplasm where their diffusion is hindered by internal membranes in the cytoplasm (Figures S13 and S15). These findings are in line with experimental findings reported in the literature and with theoretical findings showing that the cytoplasm behaves to a very large extent as a liquid phase for length scales shorter than 100 nm and as a dynamically structured macromolecular matrix for longer length scales.^63^ They are also important for the validation of our instrument performance.

Importantly, the integrated mpFCS/FLIM system enabled us to characterize in live cells the heterogeneous reaction-diffusion landscape of transcription factor OLIG2-eGFP and provided important new insights into its intracellular organization. It also enabled us to characterize in great detail the effects of the allosteric inhibitor NSC 50467 on OLIG2-eGFP homodimerization and interactions with chromatin DNA. The possibility to quantitatively characterize in live cells location-specific differences in transcription factor concentration, homodimerization, and DNA binding and the effect of pharmacological agents on these determinants of transcription factor function opens transcription factors to experimental therapeutics. Here, we have shown that the therapeutic compound NSC 50467 targeting OLIG2 homodimerization efficiently abolishes OLIG2-eGFP binding to chromatin DNA. We have also shown that this compound does not affect OLIG2-eGFP levels in the cytoplasm and its distribution in cytoplasmic organelles/membrane-less microdomains. The possibility to perform such detailed, previously intractable measurements may significantly facilitate new therapeutic discoveries.

In conclusion, the methodology presented here is a versatile tool with myriads of applications in biomedical research. In its current realization with 256 (16 × 16) OVEs, simultaneous sampling in cellular organelles is limited to a handful of locations. This, however, can be improved using another DOE (e.g., (32 × 32), as we have previously shown^35,39^). Also, while we have demonstrated the application of our method for studies in live tissue *ex vivo,*(35,39) our approach is better suited for studies in cell cultures, where the background from scattered fluorescence is lower than in tissues/small organisms. Despite these limitations, the strength of our approach lies in the user-friendly instrument design and the capacity of our methods to characterize both, compartmentalization of molecular processes, by measuring local excited-state decay via FLIM, and their dynamic integration, by measuring diffusion/active transport using mpFCS. Compartmentalization and dynamic integration of molecular processes are opposed yet coexisting and intertwined principles essential for normal cellular physiology as they enable location-specific processing of information and integral whole-cell response. Our methodology is thus paving the way to better understanding how biological functions emerge from underlying spatially confined chemical processes.

## Supporting Information Available

S1: Optical setup for mpFCS/FLIM, data analysis, and image rendering; S2: Cell culture and transfection procedures; S3: Determining the apparent dissociation constant of OLIG2-eGFP homodimers; S4: Determining the apparent dissociation constant for OLIG2-eGFP binding to chromatin DNA by FCS; and S5: Standard solutions for mpFCS/FLIM system calibration (PDF)

## Notes

Research reported in this publication was supported by the National Institute on Alcohol Abuse and Alcoholism of the National Institutes of Health under Award Number R01AA028549. The content is solely the responsibility of the authors and does not necessarily represent the official views of the National Institutes of Health. The funding agencies had no influence on the study design, methods, data collection, analyses, or the manuscript writing. The authors declare no competing financial interest. All data are available in the main text or the supporting materials.
