# Supplementary material for: Dynamic Cellular Cartography: Mapping the Local Determinants of Oligodendrocyte Transcription Factor 2 (OLIG2) Function in Live Cells Using Massively Parallel Fluorescence Correlation Spectroscopy Integrated with Fluorescence Lifetime Imaging Microscopy (mpFCS/FLIM)
Source: Anal Chem. 2021 Aug 24;93(35):12011–21. doi: 10.1021/acs.analchem.1c02144 (PMC8427561; doi:10.1021/acs.analchem.1c02144)
Supplement: Supplementary file 1 — ac1c02144_si_001.pdf [file ac1c02144_si_001.pdf]

Supporting Information: Dynamic Cellular Cartography: Mapping the local determinants of oligodendrocyte transcription factor 2 (OLIG2) function in live cells using massively parallel Fluorescence Correlation Spectroscopy integrated with Fluorescence Lifetime Imaging Microscopy (mpFCS/FLIM)

Sho Oasa[1,‡], Aleksandar J. Krmpot[1,2,‡], Stanko N. Nikolić[1,2,‡], Andrew H. A. Clayton[3], Igor F. Tsigelny[4], Jean-Pierre Changeux[5], Lars Terenius[1], Rudolf Rigler[1,6,*], Vladana Vukojević[1,*]

[1]Department of Clinical Neuroscience (CNS), Center for Molecular Medicine (CMM), Karolinska Institutet, 17176 Stockholm, Sweden. [2]Institute of Physics Belgrade, University of Belgrade, 11080 Belgrade, Serbia. [3]Optical Sciences Centre, Department of Physics and Astronomy, School of Science, Swinburne University of Technology, Melbourne, Victoria 3122, Australia. [4]Department of Neurosciences, University of California San Diego, La Jolla, California 92093-0819. [5]Department of Neuroscience, Unité Neurobiologie Intégrative des Systemes Cholinergiques, Institut Pasteur, F-75724 Paris 15, France. [6]Department of Medical Biochemistry and Biophysics (MBB), Karolinska Institutet, 17177 Stockholm, Sweden.

SUMMARY: (S1) Optical setup for mpFCS/FLIM, data analysis and image rendering. **S1a:** Optical setup and alignment; **S1b:** mpFCS data acquisition, analysis and instrument calibration; **S1c:** FLIM measurement, data analysis and image rendering; **S1d:** Phasor analysis of FLIM; **S1e:** FLIM in complex sample. **(S2) Cell culture. S2a:** Imaging; **S2b:** Pharmacological treatment; **S2c:** FLIM-FRET. **(S3)** Determining the concentration of OLIG2-eGFP homodimers based on molecular brightness. **(S4)** Determining the apparent dissociation constant for OLIG2-eGFP binding to chromatin DNA. **(S5)** Standard solutions for calibration.

S1. OPTICAL SETUP FOR MASSIVELY PARALLEL FLUORESCENCE CORRELATION SPECTROSCOPY INTEGRATED WITH FLUORESCENCE LIFETIME IMAGING MICROSCOPY (mpFCS/FLIM), DATA ANALYSIS AND IMAGE RENDERING

S1a: mpFCS/FLIM instrumental setup. Principal components that comprise the mpFCS/FLIM system shown in Fig. 1A in the main text are: (1) a directly current-modulated picosecond 482 nm laser switchable between continuous wavelength (CW) or pulsed mode (Laser Diode Head LDH-D-C-485 driven using the Picosecond Pulsed Diode Laser Driver PDL 800-D for Pulsed and CW operation, PicoQuant, Germany); (2) a 16×16 Diffractive Optical Element (DOE; Holoeye Photonics AG, Germany); (3) a photon-counting camera based on a 64×32 Single-Photon Avalanche Diode (SPAD) array (SPC^3 Single Photon Counting Camera, Micro Photon Devices MPD, Italy), where the photosensitive area of the chip consists of 64×32 circular SPADs that are 30 µm in diameter and the distance between adjacent diodes along a row/column, *i.e.*, the pitch of the camera is 150 µm; and (4) an objective (C-Apochromat 63×/1.2 W Corr) mounted on an inverted epi-fluorescence microscope Axio Observer Z1 equipped with a high efficiency filter set (Filter Set 38 HE) for enhanced Green Fluorescent Protein (eGFP) that consists of an excitation bandpass filter EX BP 470/40 nm (central wavelength/ bandwidth), long pass dichroic mirror with a cutoff wavelength of 495 nm, and an emission band pass filter EM BP 525/50 (all from Carl Zeiss, Germany). These components are assembled on an optical table with active vibration damping (Thorlabs Inc., USA) and connected using standard opto-mechanical components (Newport Corporation, USA, and Thorlabs Inc., USA). An anamorphic antireflection coated prism pair (PS879-A, Thorlabs Inc., USA) is used to correct the intrinsic ellipticity of the laser beam, (3:1 ratio of major to minor axis); and the laser beam is further shaped and expanded by a Kepler telescope setup (L1 and L2) with a 15 µm pinhole in its focus to obtain a laser beam with a circular cross-section (15 mm) and a Gaussian intensity distribution profile. The expanded Gaussian laser beam is focused by a focusing lens (L3) and diffracted by the DOE to generate 16×16 foci in the image plane of the back port of the microscope. The illumination matrix is imaged by the microscope relay optics (schematically presented by L4) and the objective lens to the object plane. Fluorescence is collected with the same objective lens and imaged either using a Complementary Metal Oxide Semiconductor (CMOS) 18.0 Megapixel Digital Single-Lens Reflex (DSLR) camera Canon EOS 100D, pixel size 18.5 µm^2 and pixel pitch of 4.3 µm (Canon Inc., Japan), used to enable fast sample localization, or the 64×32 SPC^3 SPAD camera used for time-resolved measurements. The light path between the two camera ports was manually switched.

A uniform thin fluorescence layer deposited on the coverslips surface by a highlighter marker was used for checking daily the optical alignment of the mpFCS/FLIM system.

S1b: mpFCS measurement, data analysis and image rendering. Raw mpFCS data, *i.e.* photon counts, were acquired using the so-called free-running operating mode of the SPC^3 camera; stored on the camera's internal memory and then transferred to an SSD storage unit in a HP Workstation Z440-Xeon E5-1620 3.5 GHz using the high-speed USB 3.0 computer interface that the SPC^3 camera is equipped with. Fast data analysis by auto- and cross-correlation was performed using an NVIDIA GeForce GTX 780 graphic card containing 2304 Compute Unified Device Architecture (CUDA) cores as describe in detail in Krmpot *et al.*[1].

For mpFCS measurements in solution, fluorescence intensity fluctuations were acquired for 10 s with a temporal resolution of 10 µs *per* frame. mpFCS measurements in live cells lasted 20 s, with a temporal resolution of 20 µs *per* frame. To map the concentration, the amplitude of the autocorrelation curves (ACCs) simultaneously recorded in 16×16 observation volume elements (OVEs) was estimated from the value of G(τ) at lag time τ = 10 µs, G(10 µs), for measurements in solution and G(τ) at lag time τ = 20 µs, G(20 µs), for measurements in live cells. The average number of molecules in the OVE calculated from the autocorrelation amplitude as N = 1/(G(10 µs) – 1) or N = 1/(G(20 µs) – 1). To render diffusion time (τ_D) maps, the characteristic decay time of the ACCs was determined from its full width at half maximum. If not otherwise indicated, the full width of the ACC at half maximum is plotted in the fFMI images to show the spatial distribution of average translational diffusion times (τ_D), respectively.ACCs were fitted using the analytical function for three-dimensional (3D) diffusion of one (i = 1) or two (i = 2) components and, where appropriate, triplet formation:

$$G(\tau) = 1 + \frac{1}{N} \cdot \left[1 + \frac{T}{1-T} e^{-\frac{\tau}{\tau_T}}\right] \cdot \left(\sum_{i=1}^{n} \frac{f_{D,i}}{\left(1 + \left(\frac{\tau}{\tau_{D_i}}\right)^{\alpha}\right) \sqrt{1 + \frac{\omega_{xy}^2}{\omega_z^2} \left(\frac{\tau}{\tau_{D_i}}\right)^{\alpha}}} \right) \quad \text{(S1)}$$

In equation (S1), N is the average number of molecules in the OVE; T is the average equilibrium fraction of molecules in the triplet state (when not applicable, T = 0); and τ_T is the triplet correlation time, related to the rate constants for intersystem crossing and the triplet decay; f_i is the relative amplitude of the i-th component and the sum of all relative amplitudes is equal to 1, $\sum_{i=1}^{n} f_{D,i} = 1$; τ_{Di} is the translational diffusion time of the i-th component; ω_{xy} and ω_z are the $1/e^2$ radial and axial radii of the observation volume element (OVE), respectively; α is the anomalous diffusion exponent: α = 1, for free 3D diffusion; α < 1 for sub-diffusion, *e.g.* as a result of molecular crowding – the smaller α, the greater the crowding; or α > 1 for super-diffusion, *e.g.* as a result of active cellular transport. The diffusion coefficient D was determined from the diffusion time using the relationship $\tau_D = \omega_{xy}^2/D$. Offline ACCs fitting was performed using the Origin Data Analysis and Graphing Software (OriginLab Corporation, USA).

The local molar concentration was determined as $c_{local\,(l,m)} = N_{l,m}/(N_A \cdot V_{l,m})$, where N_A is the Avogadro's number (6.02×10^{23} mol^{-1}), $N_{l,m}$ is the average number of molecules in the individual (l,m) OVE determined by fitting the corresponding experimental ACC, and $V_{l,m}$ is the volume of the (l,m) observation volume element (OVE).

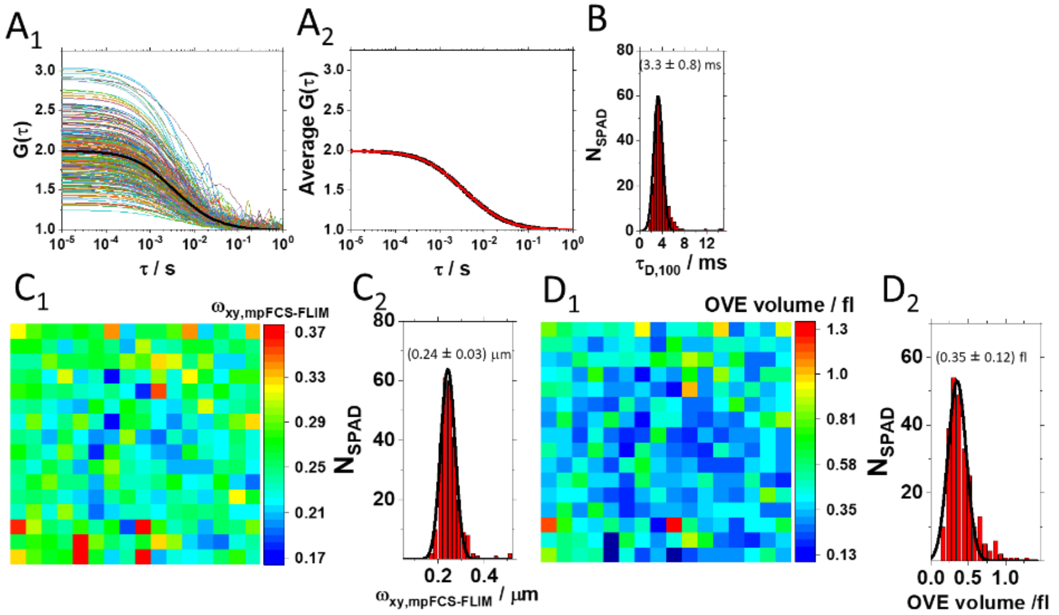

Fig. S1. Characterizing the Observation Volume Element (OVE) size in the integrated mpFCS/FLIM system using 100 nm fluospheres. **(A_1)** 256 single-SPAD autocorrelation curves (ACCs) recorded in an aqueous suspension of 100 nm fluospheres, c = 2 nM (by spFCS), with the corresponding average ACC (black). **(A_2)** Fitting the analytical function for three-dimensional (3D) diffusion of one component (red) to the average ACC shown in (A_1; black). The average structure parameter was determined to be s = 4.6. **(B)** Histogram of diffusion times determined from the full width at half-maximum of the ACCs shown in (A_1). From the best-fit Gaussian curve (black), the average diffusion time was determined, $\tau_{D,100}$ = (3.3 ± 0.8) ms. **(C_1)** Spatial map of OVE waist radius ($\omega_{xy,mpFCS\text{-}FLIM}$) derived from the diffusion times in (B) and the diffusion constant of 100 nm fluospheres, D_{100} = 4.4 µm²/s, using equation: $\tau_D = \omega_{xy}^2/4D$. **($C_2$)** Corresponding histogram of OVE waist radius. From the best-fit Gaussian curve (black), the average OVE waist radius was determined, $\omega_{xy,mpFCS/FLIM}$ = (0.24 ± 0.03) µm. **(D_1)** Spatial map of the effective OVE volume ($V_{OVE,mpFCS/FLIM}$), derived from the waist radius in (C_1) and the average structure parameter s = 4.6, using equation: $V_{OVE} = \pi^{3/2}\omega_{xy}^3 s$. **($D_2$)** From the best-fit Gaussian curve (black), the average effective OVE volume was determined to be $V_{OVE,mpFCS\text{-}FLIM}$ = (0.35 ± 0.12) fl.Data on mpFCS/FLIM system calibration for mpFCS measurements are shown in Figs S1-S5.

To begin with, the observation volume element (OVE) size was measured using 100 nm fluospheres (diameter d = 100 nm; diffusion coefficient D_{100} = 4.4 μm^2 s^{-1})[2] (Fig. S1).

Single-SPAD autocorrelation curves (ACCs) acquired in a 2 nM aqueous suspension of fluospheres, the concentration of which was determined using the reference single point FCS (spFCS) system, are shown in Fig. S1A$_1$. By fitting the average ACC using a theoretically derived equation for free 3D diffusion of a single component (eq. (S1), α = 1, i = 1, T = 0; Fig. S1A$_2$, red), the average diffusion time (τ_D), $\tau_{D,100}$ = (3.3 ± 0.8) ms (Fig. S1B); the average structure parameter s = 4.6; and the average OVE waist radius $\omega_{xy,mpFCS/FLIM}$ = (0.24 ± 0.03) μm (Fig. S1C), were determined. The $\omega_{xy,mpFCS/FLIM}$ value is similar to the OVE radius measured in the reference spFCS system, $\omega_{xy,spFCS}$ = (0.25 ± 0.04) μm, determined using a dilute solution of ATTO488 (data not shown). Using the experimentally determined average structure parameter (Fig. S1A$_2$) and the OVE waist radius (Fig. S1C), the effective volume, $V_{OVE,mpFCS\text{-}FLIM}$ = (0.35 ± 0.12)×10^{-18} m^3 and the average axial radius of the OVEs was determined, $\omega_{z,mpFCS/FLIM}$ = $\omega_{xy,mpFCS/FLIM}\cdot s$ = 0.24 μm · 4.6 = (1.1 ± 0.2) μm.

The mpFCS/FLIM system calibration was further verified using an aqueous suspension of quantum dots (Fig. S2).

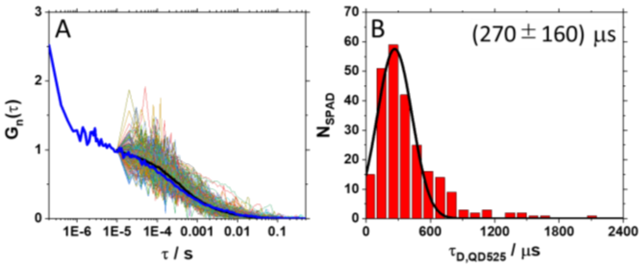

Fig. S2. Performance of the integrated mpFCS/FLIM system compared to the conventional spFCS system using an aqueous suspension of quantum dots. (A) 256 single-SPAD autocorrelation curves (ACCs) normalized to the same amplitude, G(τ) = 1 at lag time τ = 10 μs, recorded in an aqueous suspension of quantum dots (QD525), c_{QD525} = 7 nM, and the corresponding average ACC determined by mpFCS (black). ACC recorded in the same suspension using the conventional single-point FCS (spFCS) used as a reference (blue). **(B)** Histogram of diffusion times determined from the full width at half-maximum of the ACCs shown in (A). From the best-fit Gaussian curve, the average diffusion time was determined, $\tau_{D,QD525}$ = (270 ± 160) μs. In some pixels, significantly longer diffusion times, 600 μs – 2 ms, were measured likely to be due to the formation of QD525 agglomerates.

A 4 nM buffered aqueous solution of the enhanced Green Fluorescent Protein (eGFP) was used to confirm single-molecule sensitivity of the mpFCS/FLIM system (Fig. S3) and assess whether possible artifacts are arising due to deviations of the OVE shape from a 3D-ellipsoidal Gaussian (Fig. S4A and S4B). To this aim, goodness of fitting the average ACC recorded in the aqueous buffer solution of eGFP (c_{eGFP} = 4 nM) using the equation for a one-component free 3D diffusion in a Gaussian OVE (eq. (S1), α = 1, i = 1, T = 0) was examined[3]. As can be seen (Fig. S4A and S4B), a poor fit with large fitting residuals at long time scales and a divergent axial ratio, which are indications of a non-Gaussian observation volume, were not observed, indicating that the assumption of a Gaussian observation profile is valid.

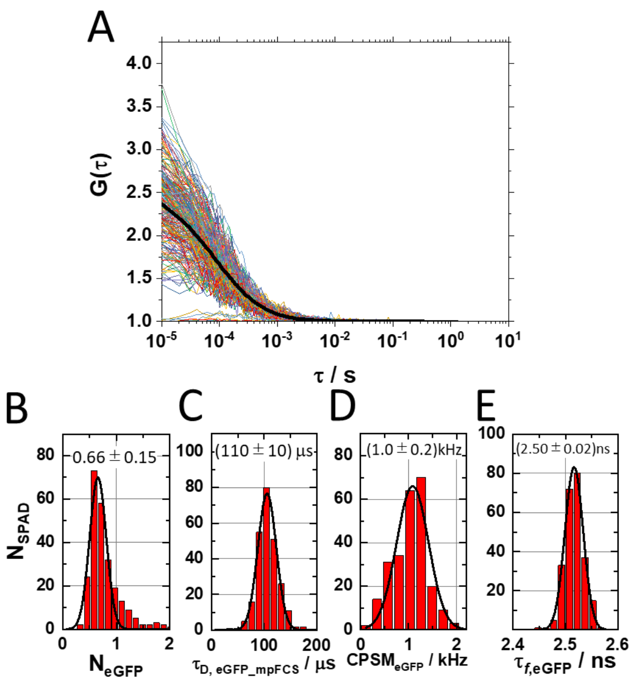

Fig. S3. Measuring with single-molecule sensitivity the concentration, diffusion, brightness and fluorescence lifetime of eGFP in an aqueous phosphate buffer solution using the integrated mpFCS/FLIM system. (A) 256 single-SPAD autocorrelation curves (ACCs) recorded in an aqueous buffer solution of eGFP, c_{eGFP} = 4 nM, with the corresponding average ACC (black). **(B)** Histogram of number of eGFP molecules, determined from the ACCs shown in (A). From the best-fit Gaussian curve (black), the average ACC amplitude was determined, N_{eGFP} = (0.66 ± 0.15). **(C)** Histogram of diffusion times determined from the full width at half-maximum of the ACCs shown in (A). From the best-fit Gaussian curve (black), the average diffusion time was determined, $\tau_{D,eGFP,mpFCS}$ = (110 ± 10) μs. **(D)** Histogram of eGFP brightness (CPSM). From the best-fit Gaussian curve (black), the average eGFP brightness was determined, $CPSM_{eGFP}$ = (1.0 ± 0.2) kHz. **(E)** Histogram of eGFP fluorescence lifetimes. From the best-fit Gaussian curve (black), the average eGFP fluorescence lifetime was determined, $\tau_{f,eGFP}$ = (2.50 ± 0.02) ns.

Finally, the axial resolution (Fig. S4C) and sensitivity of the integrated mpFCS/FLIM system were characterized (Fig. S5).

The axial resolution of the mpFCS/FLIM system was assessed in two ways, from FCS calibration experiments using a 2 nM aqueous suspension of fluospheres (Fig. S1), and z-stack imaging of a thin fluorescence layer deposited on the coverslip surface by a highlighter marker (Fig. S4C, black symbols). As described above, FCS calibration experiments yielded the axial radius $\omega_{z,mpFCS/FLIM}$ = (1.1 ± 0.2) μm, in good agreement with the value derived from z-stack imaging, for which the best-fit Gaussian curve of the fluorescence intensity profile as a function of distance from the focal plane yielded the half width at half maximum, HWHM = (1.15 ± 0.09) μm (Fig. S4C, red solid line).

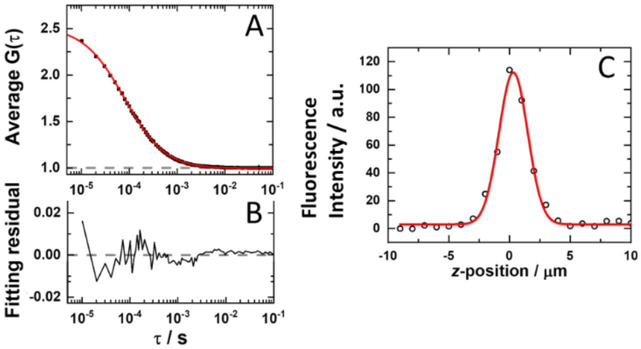

Figure S4. Assessing possible deviation of the observation volume element (OVE) shape from a 3D-ellipsoidal Gaussian and the Axial resolution of the mpFCS/FLIM system. (A) Average ACC recorded in an aqueous buffer solution of eGFP, c_{eGFP} = 4 nM (black symbols; same data as shown in Fig. 1D$_1$ and Fig. S3A) fitted using a one-component free 3D diffusion model in a Gaussian OVE (eq. (S1); α = 1, i = 1, T = 0). **(B)** Residuals corresponding to data shown in (A). **(C)** Fluorescence intensity as a function of distance from the focal plane.

The sensitivity of the integrated mpFCS/FLIM system was characterized using standard dilution series (Fig. S5).

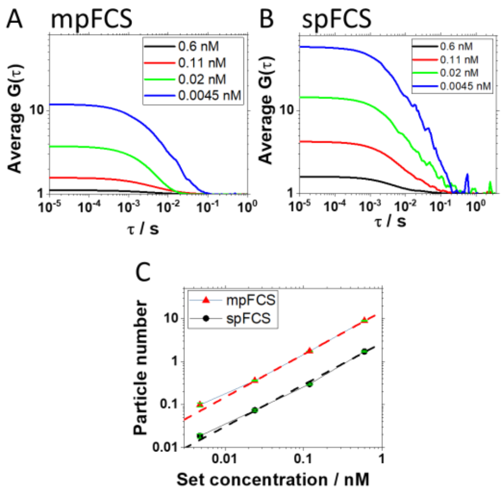

Figure S5. Sensitivity of the integrated mpFCS/FLIM system compared to that of the conventional spFCS system assessed using a dilution series of 100 nm fluospheres in aqueous suspensions. (A) Average autocorrelation curves (ACCs) recorded by the integrated mpFCS/FLIM system in a series of diluted suspensions of 100 nm fluospheres in water. The indicated nominal concentrations, calculated from the concentration of the original solution as provided by the manufacturer, were: 0.6 nM (black), 0.11nM (red), 0.02 nM (green) and 0.0045 nM (blue). **(B)** Average ACCs acquired on the same samples as in (A) using the reference spFCS system. **(C)** Particle number determined by mpFCS (red) and spFCS (black) as a function of the nominal concentration of the fluospheres in a suspension. Linear regression analysis revealed that the average particle concentration determined by mpFCS was 5-fold higher than the concentration determined by spFCS. SDs are shown as yellow and green error bars.

S1c: FLIM measurement, data analysis and image rendering using the integrated mpFCS/FLIM system. For fluorescence lifetime measurements, the so-called time-gating operating mode of the SPC^3 camera was used[1, 4]. To this aim, the laser is operated in the pulsed mode, pulse repetition rate 50 MHz, with a pulse being triggered by the SPAD camera, after which the gate is opened. The gate width (smallest available value 220 ps) and step duration (smallest available value 20 ps) were adjusted to provide optimal trade-off between signal-to-noise; fluorescence lifetime accuracy, taking into account the convolution of the Instrumental Response Function (IRF) with the intensity measured at the first time gate (Fig. S6), and signal acquisition time. The number of steps was adjusted to match the time frame of the 20 ns internal clock following instructions provided in the MPD SPC^3 User Manual (http://www.micro-photon-evices.com/MPD/media/UserManuals/SPC3_usermanual.pdf).

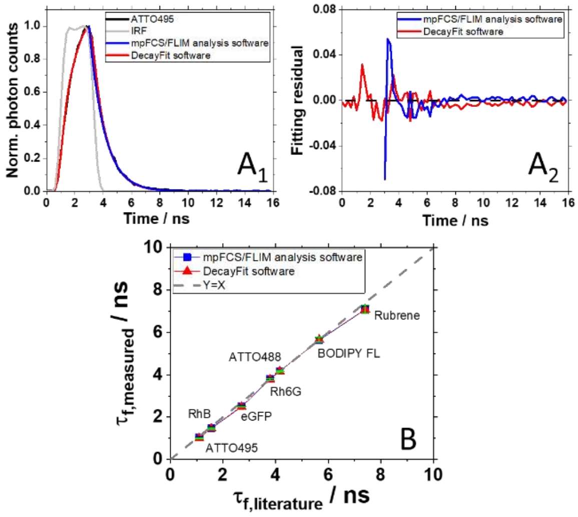

Figure S6. Effect of Instrumental Response Function (IRF) on fluorescence lifetime measurement using the integrated mpFCS/FLIM system. (A$_1$) Single-SPAD FLIM curve recorded in an aqueous solution of ATTO495, $c_{ATTO495}$ = 100 nM (black). Fluorescence lifetime of ATTO495 is $\tau_{f,ATTO495,literature}$ = 1.10 ns. Fitting of one-component exponential decay function to this FLIM curve using our mpFCS/FLIM software (blue), yields $\tau_{f,ATTO495}$ = 1.05 ns. Convolution fitting with IRF (red) using the freely available DecayFit software (http://www.fluortools.com/software/decayfit), yields $\tau_{f,ATTO495}$ = 1.00 ns. **(A$_2$)** Residuals between observed and fitted photon counts. Both models had similar residuals plot at times longer than 1 ns from the excitation pulse, but the convolution fit (red) exhibited fluctuations at earlier times than the simple mono-exponential decay fit without IRF deconvolution (blue). **(B)** Comparison between fluorescence lifetimes of several standard dyes, 1.0 ns ≤ τ_f ≤ 8 ns, measured using the integrated mpFCS/FLIM system and determined by fitting a simple mono-exponential decay curve (blue symbols) to the FLIM fluorescence decay curve or by using the convolution fit with IRF (red symbols). The dashed grey line shows the perfect correlation between experimental and literature values. SDs are shown as green error bars.

To optimize the gate width and step duration, ATTO488 in aqueous buffer solution was used as a fluorescence lifetime standard, $\tau_{f,ATTO488}$ = 4.16 ns. Gate widths and step durations were chosen based on the appearance of the fluorescence decay curves (Fig. S7) and the accuracy and precision of the measured lifetimes relative to the lifetime standard (Fig. S6). (Of note, curves that were noisy or had spurious fluctuations, *e.g.* Fig. S7A, black, were rejected.)

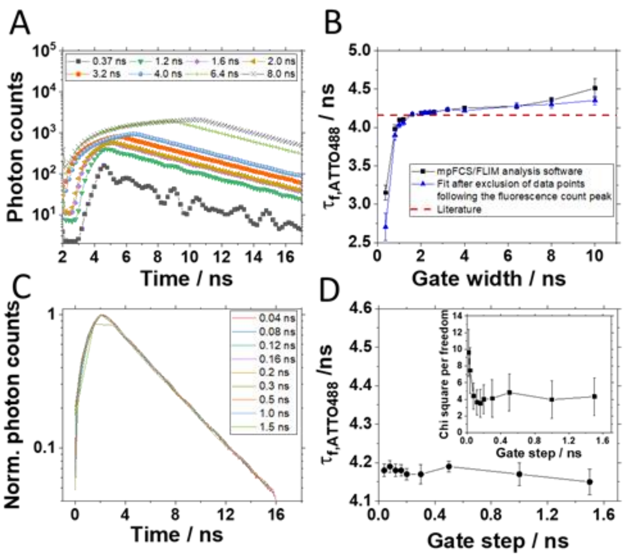

Figure S7. Effect of gate width and gate step time on measured fluorescence lifetimes. (A) Single-SPAD FLIM curves recorded in an aqueous solution of ATTO488, $c_{ATTO488}$ = 100 nM, acquired using different gate widths, 0.37 ns – 10 ns. Note the similarity in the intensity decays for gate widths 1.2 ns – 4 ns. **(B)** Effect of gate width on the extracted fluorescence lifetime. The mpFCS/FLIM data analysis software performs fitting starting from the 3rd data point after the photon count peak. Extracted fluorescence lifetimes increased for increasing gate widths, because the IRF effect prolonged to longer times for longer gate widths. To verify this interpretation, single-exponential decay fitting was performed after excluding more and more data points from the photon counts peak as the gate width was increased. While the agreement between measured and literature findings was thereby somewhat improved for longer gate widths (8.0 ns and 10 ns), the agreement was not satisfactory. Results obtained using gate widths of 1.6 ns and 2.0 ns agreed best with literature findings, and the results obtained using a gate width of 2.0 ns were less noisy as the number of collected photons was higher than when 1.6 ns gate width was used. Therefore, 2.0 ns gate width is recommended for precise FLIM measurement. **(C)** Single-SPAD FLIM curves acquired in the same solution as in (A) using different gate step times, 0.04 ns – 1.5 ns. While similar FLIM curves were obtained for all gate step times tested, the maximum photon counts peak in the FLIM curve was missed when the 1.5 ns gate step time was used. **(D)** Fluorescence lifetime values as a function of gate step times. While similar values were obtained for all gate step times tested, the average fitting chi-square per degree of freedom decreased to a plateau for gate step times longer than 0.1 ns (inlet). Therefore, gate step time in the interval 0.12 ns – 1.0 ns is recommended for precise FLIM measurement.

Based on these criteria, we have selected to use a 2 ns gate width, a 0.2 ns step size and a total sampling of 80 steps. Thus, an individual FLIM curve, *i.e.* a fluorescence decay curve recorded by a single SPAD in the SPAD camera, consists of an array of integer photon counts (8 or 16 bits unsigned integer type data), representing the fluorescence decay curve over one FLIM period. A single value of index k in a FLIM measurement is equal to the total number of photons counted through a short gate window of 2 ns duration, $\Delta t_{\text{gate}} = 2$ ns, starting at time $t_k = t_s + (k-1)\cdot\Delta t_{\text{shift}}$ (measured from the onset of the excitation laser pulse). The entire FLIM curve is thus recorded by shifting this window by $\Delta t_{\text{shift}} =$ 0.2 ns time steps, where the first window begins at $t_s = 2$ ns start shift, the second at 2.2 ns, and so on. Eighty measurement points were recorded in total for each FLIM curve, extending over a 2-18 ns sub-period during a single 20 ns FLIM measurement time.

In order to minimize the noise and improve the precision of fluorescence lifetime measurements, mpFLIM data were acquired for 10 min (averaging over 180 FLIM curves). The single-SPAD FLIM curves acquired for all 256 SPADs, with each single-SPAD curve being an average of 180 FLIM fluorescence decay curves, are stored in a single binary file, the so-called ".spcf" file, which comprises a header and acquisition metadata followed by raw image data, which is created by the MPD Software Development Kit (SDK) functions incorporated in our program. The file format is described in the MPD SPC3 User Manual (http://www.micro-photon-evices.com/MPD/media/User Manuals/SPC3_usermanual.pdf).

Our dedicated mpFLIM data analysis software includes several options. The first option enables the user to load and graphically display any individually recorded FLIM curves for each selected pixel (i, j), where $1 \le i \le 32$ and $1 \le j \le 64$. If several FLIM measurements are acquired consecutively, the software can also calculate the arithmetic average of all FLIM curves for a given pixel, yielding an individual average FLIM curve for each pixel.

The second option enables the user to fit a mono-component exponential decay function to the experimentally derived FLIM curves:

$$y_{fit1} = y_0 + Aexp\left(-\frac{(t-t_0)}{\tau}\right) \qquad \text{(S2)}$$

or to fit an exponential decay function with two characteristic times, so-called two-component exponential decay function, to the experimentally derived FLIM curves:

$$y_{fit2} = y_0 + A_1 exp\left(-\frac{(t-t_{0_1})}{\tau_1}\right) + A_2 exp\left(-\frac{(t-t_{0_2})}{\tau_2}\right) \qquad \text{(S3)}$$

Irrespective of which fitting function is chosen, the software automatically makes initial guesses for the fitting parameters for each individual average FLIM curve. In the case of function y_{fit1}, equation (S2), the initial amplitude value A is set to the difference between the maximum and the minimum of the individual averaged FLIM curve. The time shift t_0 is simply equal to the time position of the fluorescence intensity peak, while the fluorescence lifetime τ is estimated as the time when the photon count drops e times, *i.e.* decreases to 1/e of its maximal value (measured from the FLIM peak). Offset y_0 is set to be equal to the minimal fluorescence intensity value in a FLIM curve. As for the two-component exponential decay case, the procedure for guessing the initial parameter values is similar to the one for one-component exponential decay analysis, with the exception of a procedure for guessing the fractional contribution of each process to the total fluorescence, *i.e.* for guessing the relative amplitudes A_1 and A_2 in equation (S3), for which the program assumes that: $A_1 = A_2 = A/2$.

FLIM data are further processed using the Levenberg–Marquardt's method for nonlinear least squares curve-fitting, as described in the *mrqmin* nonlinear least-squares fit, Marquardt's method, Chapter 15, Numerical Recipes 2nd ed. ANSI C Files (https://www.aquila.infn.it/pierleoni/LFC/ROUTINES/Chap15.pdf). Using our software, all fitting functions are calculated within a few seconds, which significantly increases the efficiency of the experiment. If, for any reason, the fit diverges for a particular FLIM curve, the user can select that FLIM curve, enter the initial

parameter values manually and repeat the algorithm just for the selected dataset.

S1d: Phasor analysis of FLIM data. In the phasor analysis of FLIM data, Fourier transform of FLIM fluorescence decay curves is calculated and the resulting complex number is presented in a 2D polar coordinate system where the abscissa represents the real part and the ordinate the imaginary part of the complex number[5-10]. In this way, a phase vector, so-called phasor, is obtained, for which the distance from the pole and the polar angle are uniquely defined by the fluorescence lifetime. Phasors follow the normal vector algebra and their coordinates can be added/subtracted. This allows multi-component analysis of FLIM data without the need to fit multi-component exponential decay functions to FLIM fluorescence decay curves and reduces to vector algebra the analysis of complex fluorescence intensity decays by two or more processes expected in situations where there are multiple fluorophores in a mixture or when Förster Resonance Energy Transfer (FRET) occurs between the fluorophores[5-10]. We have developed the routine for phasor analysis of FLIM data in Excel (Microsoft Office, Microsoft, Seattle, WA), using the add-in program Solver to find optimal solutions for the fitting parameters.

As a first step, Fourier transformation of the normalized FLIM fluorescence decay curve is calculated using the following equations:

$$mcos(\theta_{tot}) = \frac{\sum_0^\infty [F(t) - BG] \cdot \cos(2\pi\omega t)}{\sum_0^\infty F(t) - BG} \quad \text{(S4)}$$

$$msin(\theta_{tot}) = \frac{\sum_0^\infty [F(t) - BG] \cdot \sin(2\pi\omega t)}{\sum_0^\infty F(t) - BG} \quad \text{(S5)}$$

In equations (S4) and (S5), shown by Fereidouni *et al.*[11] to be valid for a number of time-gates that is > 16 (80 in our experiments), $F(t)$ is detected fluorescence intensity, *i.e.* photon counts at time t, BG is the background fluorescence intensity, m is the modulus, θ_{tot} is the phase angle and ω is the phasor frequency that is related in time-domain fluorescence lifetime measurements with the FLIM measurement time (T), ω = 1/T. Usually, the FLIM curve acquisition time is defined by the laser repetition frequency (here 50 MHz, yielding for a single FLIM curve a maximum measurement time of 20 ns), and a phasor frequency is conventionally chosen that matches the excitation laser repetition frequency of the pulsed laser. However, the Instrument Response Function (IRF) and the gating process effectively shorten the FLIM curve acquisition time (the actual FLIM curve acquisition time in our experiments was 13.6 ns) and alter the shape of the recorded fluorescence intensity decay curve, which manifests itself in phasor analysis through rescaling of the theoretical modulus and phasor rotating by a fixed value. To account for instrument phase delay and instrument demodulation of the signal, a series of fluorescence lifetime standards were used to determine the correction factors according to the following equations:

$$mcos(\theta_{tot})_{corr} = \frac{M_t}{M_e} \cdot [mcos(\theta_{tot}) \cdot cos(\theta_t - \theta_e) - msin(\theta_{tot}) \cdot \sin(\theta_t - \theta_e)] \quad \text{(S6)}$$

$$msin(\theta_{tot})_{corr} = \frac{M_t}{M_e} \cdot [msin(\theta_{tot}) \cdot cos(\theta_t - \theta_e) - mcos(\theta_{tot}) \cdot \sin(\theta_t - \theta_e)] \quad \text{(S7)}$$

In equations (S6) and (S7), M is the modulation, θ is the phase angle and the subscripts t and e denote the theoretical and the experimentally measured values, respectively. Using six different fluorescent dyes with known fluorescence lifetimes (Fig. S8A), the instrumental demodulation correction ratio, Mt/Me, and the instrumental phase shift correction, θt – θe, were determined Mt/Me = 1.006 and θt – θe = 0.035 rad for ω = 77 MHz (corresponding to the actual FLIM curve acquisition time of ~13 ns).

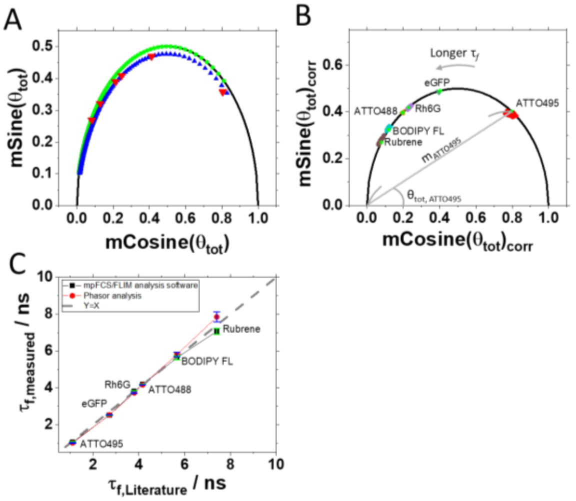

Figure S8. Phasor plot calibration and fluorescence lifetimes. (A) Phasor plot showing the universal circle (black). Phasors calculated using equations (S4) and (S5) to analyze theoretical mono-exponential decay functions with decay times from 1 ns – 20 ns (blue). Phasors calculated using equations (S11) and (S12), the phasor frequency ω = 77 MHz and fluorescence lifetime values, 1 ns ≤ τ_f ≤ 20 ns with F_2 = 0 (green). Experimentally measured uncorrected phasors (red). **(B)**. Corrected fluorescence lifetime values calculated using equations (S6), (S7) and (S8) to determine the displacement of the phasor positions. **(C)** Comparison of fluorescence lifetimes determined using single exponential fit (black rectangles) to the intensity decay data with that of the phasor approach (red circles). Dashed grey line indicates perfect agreement between the fluorescence lifetime values derived using mono-exponential fit to the FLIM fluorescence decay curves (black symbols) and the phasor analysis approach (red symbols) against literature values. SDs are shown as green error bars.

After correction, the apparent fluorescence lifetime was calculated:

$$\langle\tau\rangle = \frac{msin(\theta_{tot})_{corr}}{mcos(\theta_{tot})_{corr}} \cdot \frac{1}{2\pi\omega} \quad \text{(S8)}$$

and the phasor plot was drawn (Fig. S8B). Comparison of fluorescence lifetimes determined using one-exponential fit to the intensity decay data acquired using the phasor approach are shown in (Fig. S8C).

In a two-component mixture containing two species with different fluorescence lifetimes or in a solution of one species where fluorescence lifetime decays by two parallel processes with different half-lives, FLIM fluorescence decay curves can be described using a two-component exponential decay function:

$$F(t) = A_1\, exp\left(-\frac{t}{\tau_1}\right) + A_2\, exp\left(-\frac{t}{\tau_2}\right) \quad \text{(S9)}$$

$$f_1 = \frac{A_1}{A_1 + A_2}, \quad f_2 = \frac{A_2}{A_1 + A_2} \quad \text{(S10)}$$

where the pre-exponential factors A_1 and A_2 are amplitudes, τ_1 and τ_2 are fluorescence lifetimes, and f_1 and f_2 are the fractional contributions of species 1 and 2, respectively, to the total fluorescence.

Phasor analysis in this case yields the total phasor, which is the vector sum of the two component phasors weighted by their fractional intensity contributions. The cosine and sine components of the total phasor depend on the fractional contribution of component 1 (F_1), the lifetime of component 1 (τ_1), the lifetime of component 2 (τ_2) and the phasor frequency (ω):

$$mcos(\theta_{tot})_{corr} = \frac{F_1}{1+(2\pi\omega)^2\tau_1^2} + \frac{F_2}{1+(2\pi\omega)^2\tau_2^2} \quad \text{(S11)}$$

$$msin(\theta_{tot})_{corr} = \frac{2\pi F_1\omega\tau_1}{1+(2\pi\omega)^2\tau_1^2} + \frac{2\pi F_2\omega\tau_2}{1+(2\pi\omega)^2\tau_2^2} \quad \text{(S12)}$$

$$F_1 = \frac{A_1\tau_1}{A_1\tau_1 + A_2\tau_2}, \quad F_2 = 1 - F_1 \quad \text{(S13)}$$

To extract from FLIM fluorescence decay curves the values of these parameters, the Excel add-in program Solver was used to find optimal values along with a minimization of the chi-square value of photon counts by varying two lifetimes (τ_1 and τ_2) and the fractional contribution of component 1 (F_1):

$$\chi^2 = \frac{1}{L-p-1} \quad \text{(S14)}$$

In equation (S14), L denotes total number of gates in the FLIM curve (in our experiments L = 80); p is a parameter number; F_k is photon counts at the k-th gate in the amplitude-normalized FLIM curve, and $F_{k,phas}$ is photon counts at the k-th gate in FLIM curve re-constructed by phasor parameters.

For a two-component exponential decay, the phasor lies inside the semicircle (Fig. S9B$_3$). However, the phasor of the two-component mixture must be positioned between the phasors of the two individual components, which lie on the semicircle. The relative distance of the phasor for the mixed state from the individual components reflects the fractional intensity contribution of each component. From this, the molar fractions of the two-component mixture can be inferred.

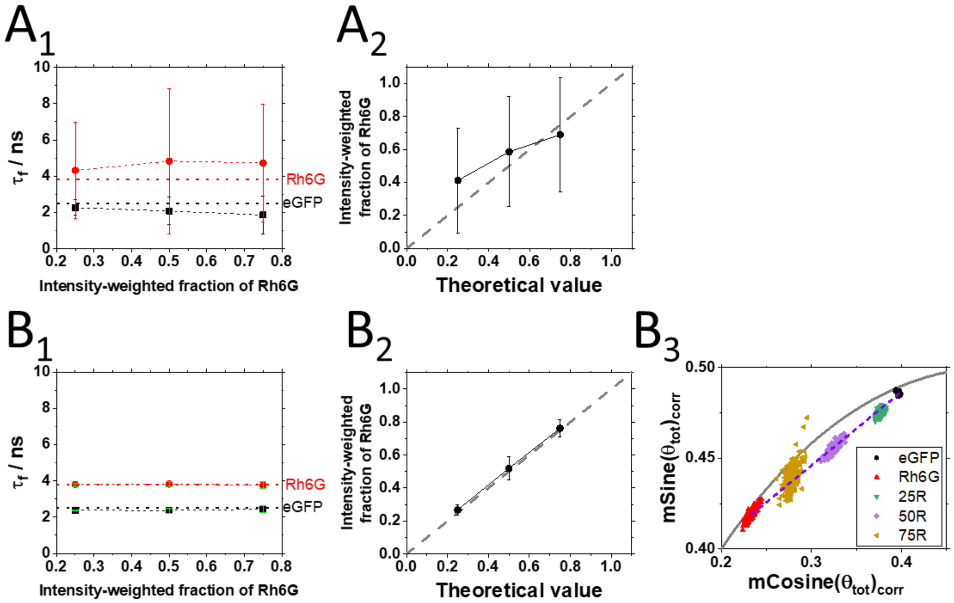

Figure S9. Fluorescence lifetime measurement in a mixture of Rh6G and eGFP, and mixture composition determination *via* mpFLIM. Comparison of results obtained by exponential decay fitting (A$_1$ and A$_2$) and phasor analysis (B$_{1\text{-}3}$). (A$_1$) Fluorescence lifetimes of Rh6G and eGFP determined using a two-component exponential decay fitting with all parameters in equation (S9) being allowed to freely vary. **(A$_2$)** Intensity-weighted fractions of Rh6G in mixtures with different Rh6G/eGFP content determined using equations (S9) and (S10) for fitting the experimentally measured mpFLIM fluorescence decay curves shown in Fig. 2C$_1$. For fitting, fluorescence lifetimes of eGFP and Rh6G were fixed to the values obtained by analysing mpFLIM fluorescence decay curves shown in Fig. 2C$_1$ (black and violet, respectively) and the intensity-weighted fraction was varied. The dashed grey line shows perfect correlation between measured and actual values. **(B$_1$)** Fluorescence lifetimes of Rh6G and eGFP determined using two-component phasor analysis, equations (S11)-(S13). SDs are shown as yellow and green error bars. **(B$_2$)** Intensity-weighted fraction of Rh6G in mixtures with different Rh6G/eGFP content determined using two-component phasor analysis, equations (S11)-(S13). The dashed grey line shows perfect correlation between measured and actual values. **(B$_3$)** Phasors in pure eGFP (black) and Rh6G (red) solutions and their mixtures containing different amounts of these components expressed as intensity-weighted fractions: Rh6G:eGFP = 0.25:0.75 (green), 0.5:0.5 (lilac), 0.75:0.25 (ochre). Of note, phasor values measured in the mixtures are distributed along the dashed blue line connecting the eGFP (black) and Rh6G (red) phasor positions.

Finally, we have also developed a three-component phasor analysis procedure to allow characterization of OLIG2 dimerization by FLIM-FRET using eGFP and ShadowY (ShY) as FRET donor and acceptor, respectively. eGFP and ShY constitute a suitable donor-acceptor pair with a Förster radius equal to 6.2 nm[12]. However, both eGFP and ShY are efficiently excited with the 488 nm laser line (molar absorption coefficient: ε_{eGFP} = 55 900 $M^{-1}cm^{-1}$ [13] and ε_{ShY} = 136 000 $M^{-1}cm^{-1}$ [12]) and emit in the region detected by our instrumental set-up, even though ShY emission is comparatively lower due to its small fluorescence quantum yield (QY_{ShY}= 0.01[12], QY_{eGFP} = 0.6[13]). Since only a fraction of OLIG2 molecules will dimerize, there will be three characteristic decay times in cells expressing OLIG2-eGFP and OLIG2-ShY: for OLIG2-eGFP molecules that do not undergo dimerization and the eGFP donor is therefore not subject to FRET ($\tau_{f eGFP,\ donor\ only}$), for OLIG2-eGFP molecules undergoing dimerization and hence likely FRET ($\tau_{f eGFP,\ FRET}$) and for the acceptor (directly excited or indirectly excited *via* FRET, $\tau_{f ShY,\ acceptor\ only}$). To describe this situation, a three-component phasor analysis routine was developed, with fixed phasor positions of the donor (x_D, y_D) and the acceptor (x_A, y_A), determined in HEK cells expressing OLIG2 labelled with the donor or the acceptor alone, and varying along the *x*-axis the phasor position of the FRET component (x_F):

$$x_{tot,corr} = F_D \cdot x_D + F_A \cdot x_A + F_F \cdot x_F \quad \text{(S15)}$$

$$y_{tot,corr} = F_D \cdot y_D + F_A \cdot y_A + F_F \cdot y_F \quad \text{(S16)}$$

$$F_D + F_A + F_F = 1 \quad \text{(S17)}$$

$$\left(x_F - \frac{1}{2}\right)^2 + y_F^2 = \frac{1}{4} \quad \text{(S18)}$$

where, $x=m\cos(\theta)$ and $y=m\sin(\theta)$ denote phasor position for donor (D), acceptor (A) and FRET component (F). *F* is an intensity-weighted fraction of donor, acceptor and FRET component. The intensity-weighted fraction is defined by area of triangles:

$$F_D = \frac{S_D}{S_D + S_A + S_F} \quad \text{(S19)}$$

$$F_A = \frac{S_A}{S_D + S_A + S_F} \quad \text{(S20)}$$

$$F_F = \frac{S_F}{S_D + S_A + S_F} \quad \text{(S21)}$$

where, S is the area of a triangle at each diagonal position of vertices. For example, S_D is the area made by three vertexes, phasor positions of donor, acceptor and measurement in which area is defined by these phasor positions. The add-in program Solver was used to find optimal solutions for the fitting parameters along with the minimization of chi-square value as shown in equation (S14). Further information is given below in section (S2c): Positive and negative controls for phasor analysis of FRET.

S1e: FLIM in a fixed acridine orange stained section through the rhizome of the lily of the valley (Convallaria majalis). Single SPAD fluorescence intensity decay curves recorded in the fixed acridine orange stained section through the rhizome of the lily of the valley (Convallaria majalis; Fig. S10).

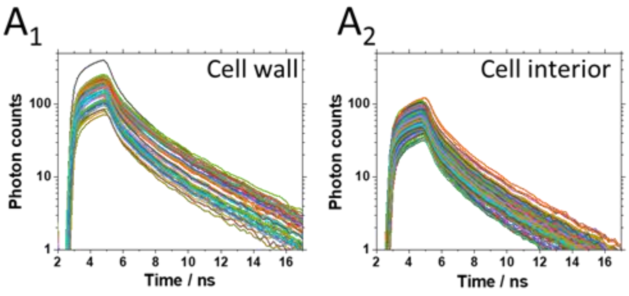

Figure S10. Single-SPAD fluorescence intensity decay curves recorded in a fixed acridine orange stained section through the rhizome of the lily of the valley (Convallaria majalis). (A_1) Single-SPAD FLIM curves recorded in the cell wall of a single cell in the central parenchyma. **(A_2)** Single-SPAD FLIM curves recorded in the interior of the same cell. Results of FLIM curve analysis are given in the main text (Fig. 3).

S2: CELL CULTURE AND PHARMACOLOGICAL TREATMENT

S2a: Cell culture and fluorescent proteins expression. HEK cells (American Type Culture Collection (ATCC)) were maintained in a humidified atmosphere containing 5 % CO_2 at 37 °C in Dulbecco's Modified Eagle Medium (Gibco Life Technologies) supplemented with 10 % fetal bovine serum (Gibco), 1 % penicillin-streptomycin (Gibco; final conc. 100 U/mL of penicillin and 100 µg/ mL streptomycin).

Expression vectors encoding the enhanced Green Fluorescent Protein (peGFP-N1), eGFP-fused OLIG2 (pOLIG2-eGFP-N1) or eGFP tetramer ($peGFP_{tet}$-C1) were transiently transfected into HEK cells [1, 14] (Figs S11-S19).

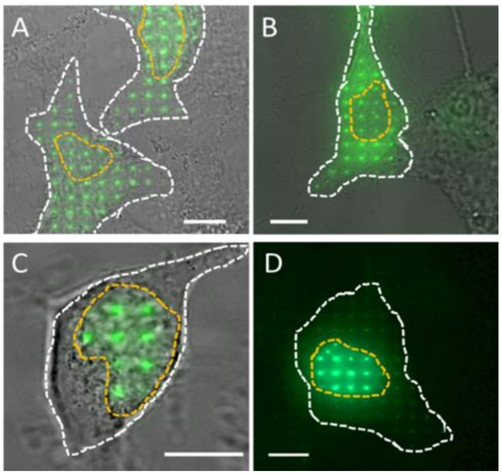

Figure S11. Fluorescence images of spot-wise (16×16) illuminated cells expressing eGFP-tagged molecules of interest (green) overlaid on a wide-field transmission image (grey) recorded using the DSLR camera. (A) Live HEK cells expressing monomeric eGFP showing its uniform distribution inside the cell. **(B)** Live HEK cells expressing the eGFP tetramer ($eGFP_{tet}$), showing preferential $eGFP_{tet}$ localization in the cytoplasm. **(C)** Live HEK cell expressing OLIG2-eGFP showing a nuclear localization of the eGFP tagged OLIG2 transcription factor. **(D)** Fluorescence image of a live HEK cell expressing OLIG2-eGFP contrasted after image acquisition to render visible the cytoplasmic pool of OLIG2-eGFP. In all images, the hand-drawn dashed lines highlight the border of the cell (white) and the cell nucleus (orange). Scale bar: 10 µm.

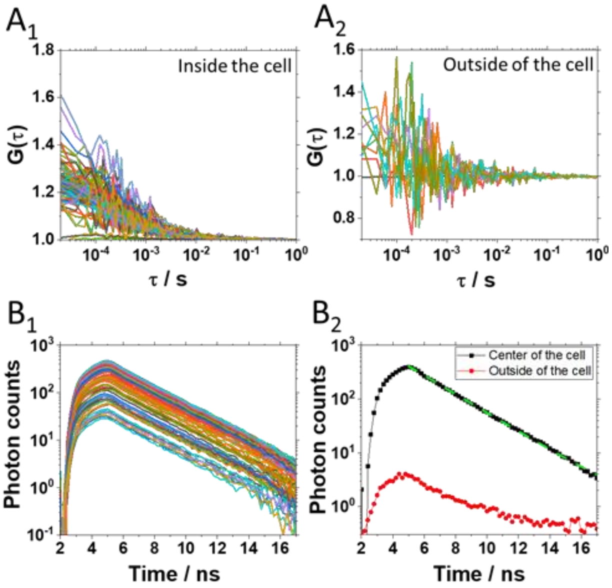

Figure S12. Single-SPAD autocorrelation curves (ACCs) and mpFLIM curves of eGFP fluorescence in a live eGFP expressing HEK cell. (A_1) Single-SPAD ACCs recorded inside the cell. **(A_2)** Single-SPAD ACCs recorded outside of the cell, in the immediately surrounding cell culture medium. **(B_1)** Single-SPAD FLIM curves recorded inside the cell. **(B_2)** A typical single-SPAD FLIM curve recorded inside the cell (black symbols) as compared to a single-SPAD FLIM curve recorded outside of the cell (red symbols), in the immediately surrounding cell culture medium. The dashed green line shows a one-exponential decay function fitted to the FLIM data recorded inside the cell.

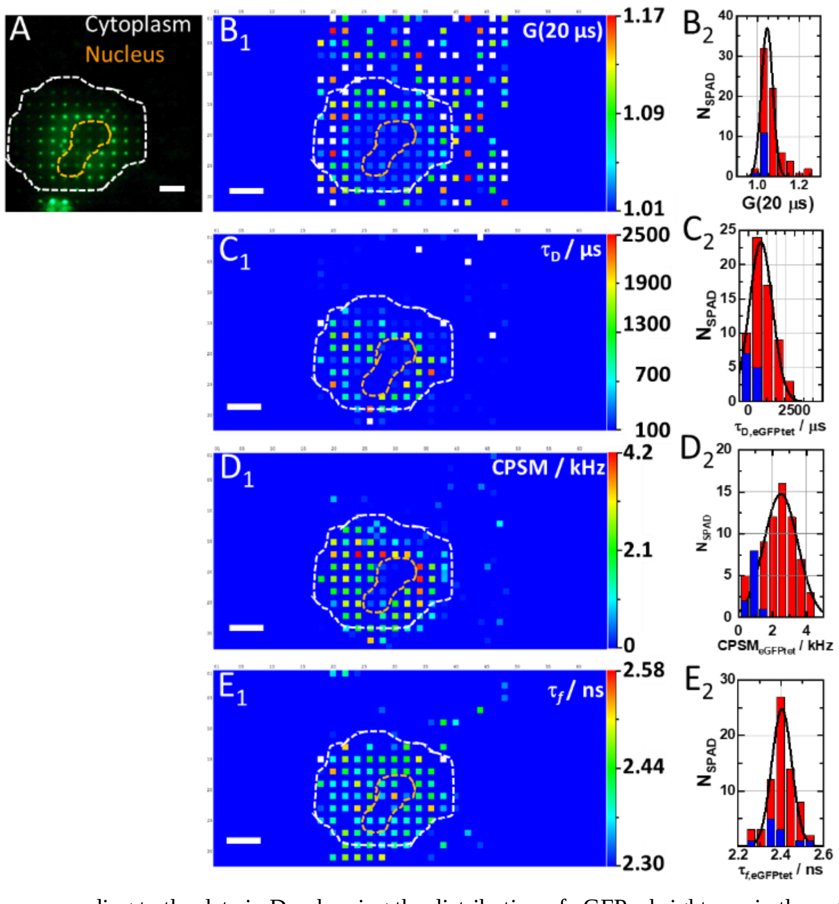

Figure S13. Spatial map of the autocorrelation amplitudes, diffusion times and fluorescence lifetimes of tetrameric eGFP ($eGFP_{tet}$) in a live HEK cell. (A) Fluorescence image of a HEK cell expressing $eGFP_{tet}$ acquired using a spot-wise, 16×16, illumination and a DSLR camera. The hand-drawn dashed lines that highlight the cell border (white) and the cell nucleus (orange), were visualized by transmission light microscopy. Corresponding ACCs and FLIM curves are shown in Fig. S14. **(B_1)** Spatial map of the autocorrelation amplitude at lag time τ = 20 µs (G(20 µs)), which is inversely proportional to the average number of eGFP tetramers in the OVE. **(B_2)** Histograms corresponding to the data in B_1, showing the distribution of autocorrelation amplitudes in the cytoplasm (red) and the nucleus (blue). From the best-fit Gaussian curve (black), the average amplitude in the cytoplasm was determined $G(20\ \mu s)_{cyt}$ = (1.05 ± 0.03), corresponding to an average concentration of $eGFP_{tet}$, $c_{eGFPtet,cyt}$ ≈ 100 nM. For ACCs recorded in the cell nucleus, please see explanation in the main text. **(C_1)** Spatial map of $eGFP_{tet}$ diffusion times. **(C_2)** Histograms corresponding to the data in C_1, showing the distribution of diffusion times in the cytoplasm (red) and the nucleus (blue). From the best-fit Gaussian curve (black), the average $eGFP_{tet}$ diffusion time in the cytoplasm was determined to be $\tau_{D,eGFPtet,cyt}$ = (700 ± 600) µs and $\tau_{D,eGFPtet,nuc}$ = (160 ± 80) µs. **(D_1)** Spatial map of $eGFP_{tet}$ brightness (CPSM). **(D_2)** Histograms corresponding to the data in D_1, showing the distribution of $eGFP_{tet}$ brightness in the cytoplasm (red) and the nucleus (blue). From the best-fit Gaussian curve (black), the average $eGFP_{tet}$ brightness was determined to be $CPSM_{eGFPtet,cyt}$ = (2.5 ± 1.0) kHz in the cytoplasm and $CPSM_{eGFPtet,nuc}$ = (0.8 ± 0.2) kHz in the nucleus. **(E_1)** Spatial map of $eGFP_{tet}$ fluorescence lifetimes. **(E_2)** Histograms corresponding to the data in E_1, showing the distribution of fluorescence lifetimes in the cytoplasm (red) and the nucleus (blue). From the best-fit Gaussian curve (black), the average $eGFP_{tet}$ fluorescence lifetime was determined to be $\tau_{f,eGFPtet,cyt}$ = (2.4 ± 0.1) ns in the cytoplasm and $\tau_{f,eGFPtet,nuc}$ = (2.4 ± 0.1) ns in the nucleus. Scale bar: 10 µm.

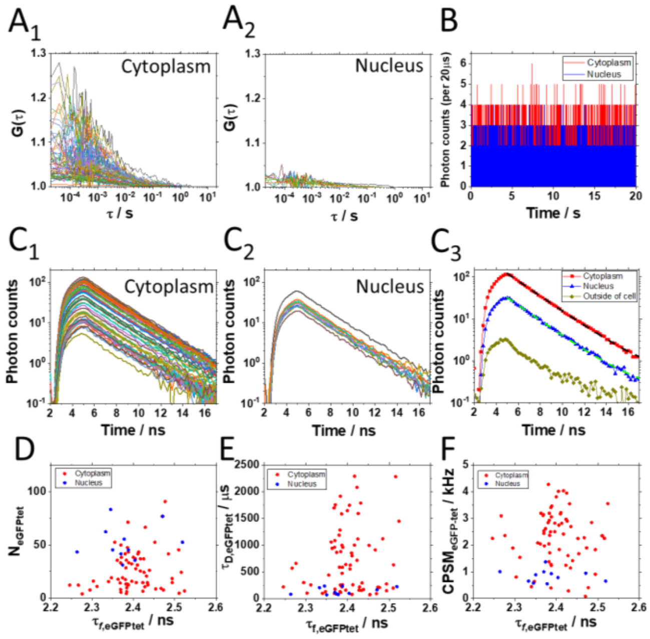

Figure S14. Single-SPAD autocorrelation curves (ACCs) and mpFLIM curves of eGFP tetramer in a live HEK cell. (A_1) Single-SPAD ACCs in the cytoplasm. **(A_2)** Single-SPAD ACCs recorded in the cell nucleus. **(B)** Typical photon counts in the cytoplasm (red) and the nucleus (blue). Due to the lower fluorescence intensity in the cell nucleus, the SNR is lower for FCS measurements in the cell nucleus. Subsequently, the amplitude of the ACCs recorded in the nucleus is lower as a consequence of the high background. **(C_1)** Single-SPAD FLIM curves recorded in the cytoplasm. **(C_2)** Single-SPAD FLIM curves recorded in the cell nucleus. **(C_3)**. A typical single-SPAD FLIM curve recorded in the cytoplasm (red symbols), cell nucleus (blue symbols) and outside the cell, in the immediately surrounding cell culture medium (ochre symbols). The dashed navy and green lines show a one-exponential decay function fitted to the FLIM data recorded in the cytoplasm and the nucleus, respectively. **(D)** The number of $eGFP_{tet}$ in the cytoplasm (red) and the nucleus (blue) against the fluorescence lifetime. **(E)** The diffusion time recorded in the cytoplasm (red) and the nucleus (blue) against the fluorescence lifetime. **(F)** The molecular brightness recorded in the cytoplasm (red) and the nucleus (blue) against the fluorescence lifetime.

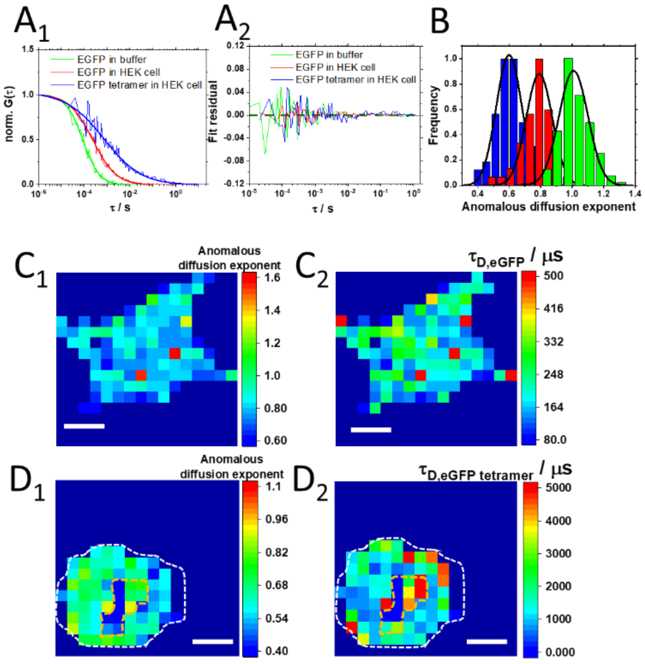

Figure S15. Anomalous diffusion analysis of eGFP and eGFP tetramer ($eGFP_{tet}$) in live HEK cells. (A_1) Typical autocorrelation curves of eGFP in phosphate buffer (green), eGFP in a live HEK cell (red), and $eGFP_{tet}$ in a live HEK cell (blue). **(A_2)** Residuals between the experimental ACCs and the fitted theoretical functions for 3D diffusion (equation (S1)) show excellent agreement (chi-square values ranging from 10^{-6} – 10^{-4}). **(B)** Histograms of the anomalous diffusion exponent distribution and average values determined from the best-fit Gaussian curves (black): $\alpha_{eGFP,buffer} = 1.00 \pm 0.09$ (green), $\alpha_{eGFP,cell} = 0.80 \pm 0.08$ (red) , and $\alpha_{eGFPtet,cell} = 0.60 \pm 0.08$ (blue). **(C_1)** Spatial map of the anomalous diffusion exponent for eGFP in a live HEK cell. The anomalous diffusion exponent in the cytoplasm is not statistically significantly different from the value measured in the cell nucleus, $p > 0.05$. **(C_2)** Spatial map of the diffusion time of eGFP in a live HEK cell. **(D_1)** Spatial map of the anomalous diffusion exponent for $eGFP_{tet}$ in a live HEK cell. The anomalous diffusion exponent in the cytoplasm, $\alpha^{cyt}_{eGFP_{tet}} = 0.6 \pm 0.1$, is statistically significantly different from the value measured in the cell nucleus, $\alpha^{nuc}_{eGFP_{tet}} = 0.5 \pm 0.3$, $p = 0.027$. **(D_2)** Spatial map of the diffusion time of $eGFP_{tet}$ in a live HEK cell. Dashed lines show nuclear region (orange) and the cell (white). Scale bar: 10 μm.

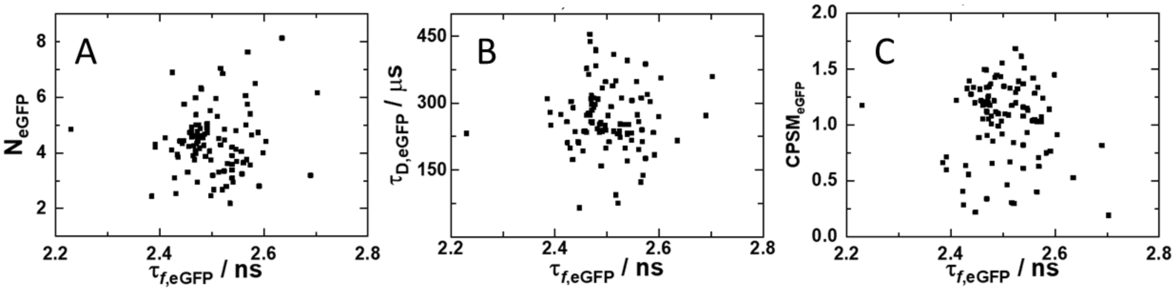

Figure S16. Scatter plots derived from spatial maps of eGFP concentration, diffusion and brightness as a function of fluorescence lifetime in a live HEK cell. (A) Scatter plots of number of eGFP against fluorescence lifetime of eGFP. **(B)** Scatter plots of eGFP diffusion time against fluorescence lifetime. **(C)** Scatter plots of eGFP CPSM against fluorescence lifetime.

S2b: Pharmacological treatment. After transfection, HEK cells were cultured for 24 h in the 8-well chambered cover glass. Thereafter, the cell culture medium was replaced either with a phenol red free medium (untreated cells) or with a phenol red free medium containing 1 µM 5-chloro-N1,N3-bis[3(4,5-dihydro-1H-imidazol-2yl)phenyl]benzene-1,3-dicarboxamide, dihydrochloride (compound NSC 50467; treated cells). Detailed information on the NSC 50467 compound pound and its preparation can be found in[14]. Briefly, the NSC 50467 compound was dissolved in Dimethyl sulfoxide (DMSO) yielding a stock solution (mM concentration) that was stored at 4 °C for future use. On the day of treatment, an aliquot was taken from the stock solution, allowed to warm up to room temperature and an adequate volume was pipetted-out and diluted with the phenol red free medium to a final concentration of 1 µM NSC 50467. After 1 h incubation, all measurements were performed at room temperature. The results are shown in Figs S17-S19.

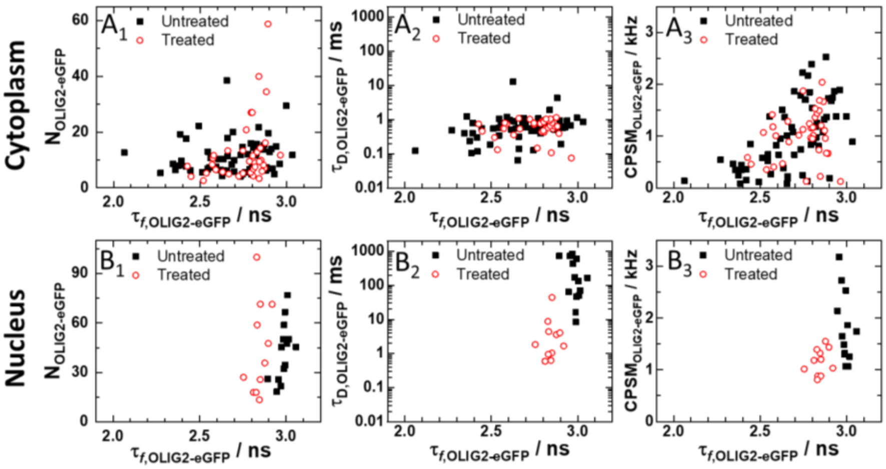

Figure S17. Relationship between number, diffusion times or brightness and fluorescence lifetime in live OLIG2-eGFP expressing HEK cells following the treatment with 1 µM NSC 50467, the allosteric inhibitor of OLIG2 homodimerization. ($A_{1\text{-}3}$) Relationship between the number (A_1), diffusion time (A_2) and molecular brightness (A_3) against fluorescence lifetime in the cytoplasm of untreated (black) and treated (red) cells. **($B_{1\text{-}3}$)** Relationship between the number (B_1), diffusion time (B_2) and molecular brightness (B_3) against fluorescence lifetime in the nucleus of untreated (black) and treated (red) cells.

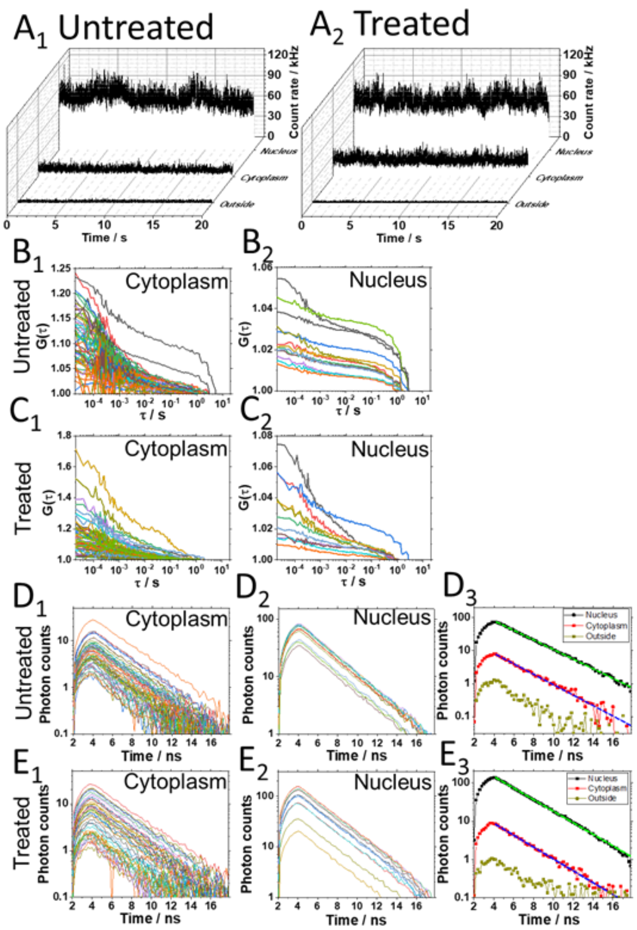

Figure S18. Single-SPAD autocorrelation curves (ACCs) and mpFLIM curves of OLIG2-eGFP in a live HEK cell before and after treatment with the allosteric inhibitor of OLIG2 homodimerization NSC 50467. (A_1) Photon counts recorded in the cytoplasm, nucleus and outside of the cell before treatment. **(A_2)** Photon counts recorded in the cytoplasm, nucleus and outside of the cell after treatment with the allosteric inhibitor of OLIG2 dimerization NSC 50467. **(B_1)** Single-SPAD ACCs recorded in the cytoplasm of an untreated cell. **(B_2)** Single-SPAD ACCs recorded in the nucleus of the same cell. **(C_1)** Single-SPAD ACCs recorded in the cytoplasm of the same cell following 1 h treatment with 1 µM NSC 50467. **(C_2)** Single-SPAD ACCs recorded in the nucleus of the same cell, following treatment with NSC 50467. **(D_1)** Single-SPAD FLIM curves recorded in the cytoplasm of an untreated cell. **(D_2)** Single-SPAD FLIM curves recorded in the nucleus of the same cell as in D_1. **(D_3)** Typical single-SPAD FLIM curves recorded in the nucleus (black symbols) and cytoplasm (red symbols) of an untreated cell and outside the cell, in its immediate surroundings (ochre symbols), together with corresponding fitted curves (dashed lines). **(E_1)** Single-SPAD FLIM curves recorded in the cytoplasm of the same cell as in (D_1) following a 1 h treatment with 1 µM NSC 50467. **(E_2)** Single-SPAD FLIM curves recorded in the nucleus of the same cell as in D_1 following treatment. **(E_3)** Typical single-SPAD FLIM curves recorded in the nucleus (black symbols) and cytoplasm (red symbols) of a treated cell and outside the cell, in its immediate surroundings (ochre symbols), together with corresponding fitted curves (dashed lines).

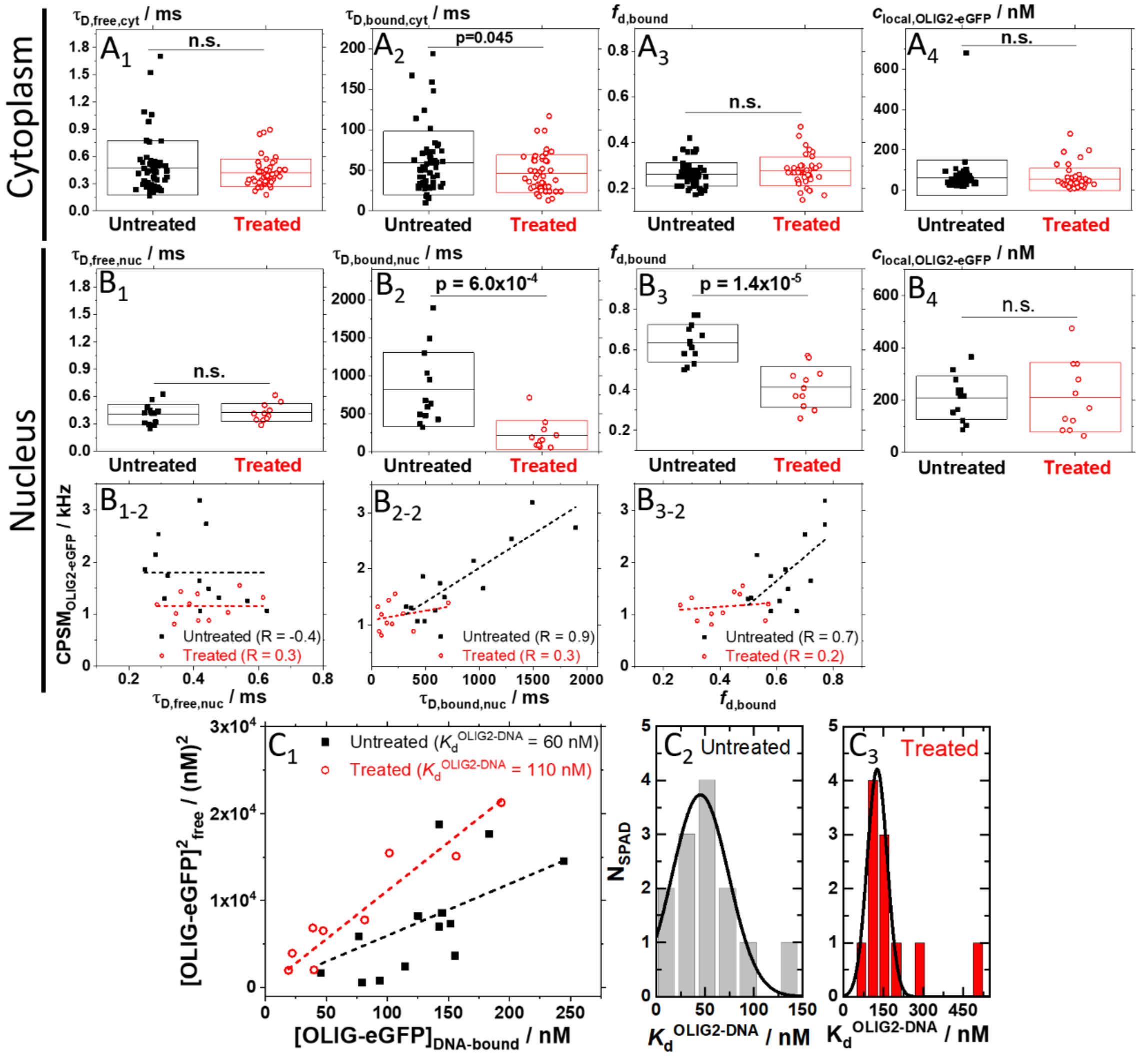

Figure S19. Determination of the apparent dissociation constant of OLIG2-eGFP binding to chromatin DNA in a live HEK cell before and after treatment with the allosteric inhibitor of OLIG2 homodimerization NSC 50467. ($A_{1\text{-}4}$) Cytoplasm: Diffusion time of free (A_1) and bound (A_2) OLIG2-eGFP, the mole fraction of bound OLIG2-eGFP (A_3) and OLIG2-eGFP local concentration in individual OVEs (A_4), before (black) and after (red) treatment. **($B_{1\text{-}4}$)** Nucleus: Diffusion time of free (B_1) and bound (B_2) OLIG2-eGFP, the mole fraction of bound OLIG2-eGFP (B_3) and OLIG2-eGFP local concentration in individual OVEs (B_4), before (black) and after (red) treatment. **($B_{i\text{-}2}$)** Molecular brightness in the nucleus, as reflected by counts *per* second and molecule (CPSM) measured in a single-SPAD, as a function of: the diffusion time of the free OLIG2-eGFP fraction ($B_{1\text{-}2}$), the diffusion time of the DNA-bound OLIG2-eGFP fraction ($B_{2\text{-}2}$) and the mole fraction of the DNA-bound OLIG2-eGFP ($B_{3\text{-}2}$) before (black) and after (red) treatment. **($C_{1\text{-}3}$)** Apparent dissociation constant of OLIG2-eGFP binding to chromatin DNA determined from linear-regression analysis of $[\text{OLIG2-eGFP}]^2_{\text{free}} = f([\text{OLIG2-eGFP}]_{\text{DNA-bound}})$ (C_1) and from Gaussian curve fitting to the histograms of apparent dissociation constant distribution before (C_2) and after treatment (C_3).

S2c: FLIM-FRET. For FLIM-FRET experiments, the expression plasmids of ShadowY (ShY) and tandem dimer of eGFP-ShY on pCPCI3 vector were kindly provided by Dr. Hideji Murakoshi, National Institute for Physiological Sciences, Aichi Japan[12]. Expression plasmids of pShY-CPCI3 and peGFP-N1 were transfected into HEK cells as a negative control, eGFP-ShY as a positive control in FRET experiments. Expression plasmid of OLIG2-ShY was constructed as previously described[14]. Briefly, OLIG2 sequence was amplified from pGEM-OLIG2 using forward primer with NheI site and reverse primer with AgeI site[15], ligating into pShY-CPCI3 vector.

One day before the transfection, HEK cells were split and transferred to 8-well chambered coverglass with 1.0×10^4 (cells/mL in each chamber). HEK cells on the 8-well chamber were transfected with 100 ng plasmid DNA and 0.2 µL Lipofectamine 2000 (Thermo Fisher Scientific) in each well. After the transfection, HEK cells were cultured for 24 h in a humidified atmosphere containing 5 % CO_2 at 37 °C. The medium was replaced to phenol red free medium, FluoroBrite™ DMEM (Gibco) just before the experiments (Figs S20 and S21).

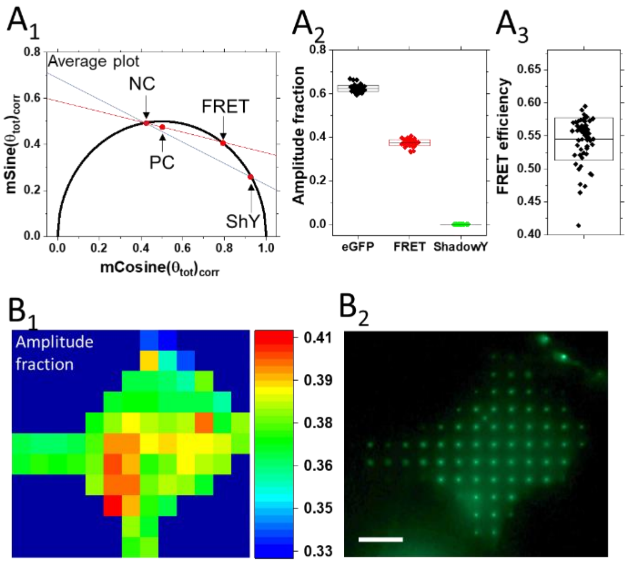

Figure S20. Positive and negative controls for phasor analysis of FRET between the eGFP donor and the ShadowY (ShY) acceptor. Negative control (NC), live HEK cells co-expressing eGFP and ShY. Positive control (PC), live HEK cells expressing the fusion protein eGFP-ShY linked *via* a peptide SGLRSG. (A_1) Average fluorescence lifetimes presented in a phasor plot. Fluorescence lifetime measured in the NC is virtually indistinguishable from the fluorescence lifetime of the eGFP donor (phasor positions for NC: (x,y) = (0.4253,0.4923) and eGFP: (x,y)=(0.4333, 0.4895)), the phasor of which is located on the semicircle, suggesting that neither FRET nor direct ShY excitation affect eGFP lifetime and its measurement in the NC cells. In contrast, the PC phasor exhibited a shift with respect to the eGFP donor, indicating a FRET contribution. **(A_2)** Amplitude of the FRET state fraction, 0.38 ± 0.02. **(A_3)** FRET efficiency in the FRET population is 0.55 ± 0.03. **(B_1)** Spatial mapping of the amplitude fraction in a PC cell shows a homogenous, tight distribution of amplitude fraction of FRET component (range: 0.33-0.41), with slightly higher amplitude fraction in some pixels. **(B_2)** Fluorescence images of a spot-wise (16×16) illuminated PC cell recorded using the DSLR camera.

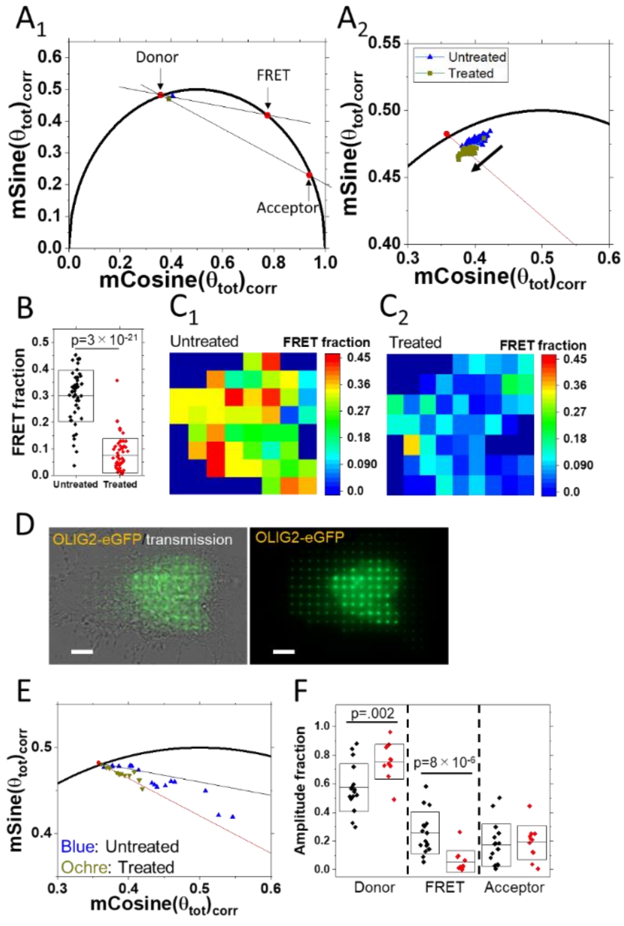

Figure S21. The effect of treatment with the allosteric inhibitor NSC 50467 on OLIG2 homodimerzation in live HEK cells co-expressing OLIG2 constructs genetically fused with the eGFP (donor) or the ShY (acceptor) characterized using the multi-component FLIM-FRET phasor analysis approach. (A_1) Phasor positions in untreated cells (blue) and cells treated with NSC 50467 (ochre) localize inside the universal circle, indicative of donor fluorescence quenching due to FRET. **(A_2)** Magnified detail of the phasor plot in (A_1) reveals that 1 h treatment with 1 μM NSC 50467 causes the phasor to move counter-clockwise, indicating less quenching due to FRET. **(B)** Amplitude fraction of the FRET component in untreated cells (0.3 ± 0.1) was reduced to (0.07 ± 0.06) upon treatment with NSC 50467, suggesting that OLIG2 dimerization is significantly reduced. **(C_1)** Spatial mapping of the amplitude fraction of FRET in untreated cells. **(C_2)** Spatial mapping of the amplitude fraction of FRET in cells treated with the inhibitory NSC 50467 compound. **(D)** *Left:* Fluorescence image of a spot-wise (16×16) illuminated HEK cell expressing OLIG2-eGFP and OLIG2-ShY (green) overlaid on a wide-field transmission image (grey) recorded using the DSLR camera. *Right:* The same fluorescence image shown on its own to better visualize the nuclear localization of OLIG2. **(E)** Positions of average phasors acquired in untreated (ochre) and treated (blue) cells. Each phasor point is an average over a single cell. The black solid line links the donor and the FRET component (FRET trajectory). The red solid line links the donor with the acceptor. Phasor plots in the absence of inhibitory compound were distributed in-between the red and black lines, suggesting OLIG2 dimer formation. On the other hand, phasor plots in the presence of inhibitory compound were shifted onto the red line, reducing FRET. **(F)** Box and whisker plot showing the amplitude fraction of each component measured in untreated (black) and treated (red) cells, suggest that the FRET fraction was significantly reduced, while the donor fraction was significantly increased, suggesting OLIG2 dimer dissociation or perturbation by inhibitory compound.

S3. DETERMINING THE CONCENTRATION OF OLIG2-EGFP HOMODIMERS BASED ON MOLECULAR BRIGHTNESS ANALYSIS BY FCS. To determine the fraction of OLIG2-eGFP homodimers based on molecular brightness analysis, the procedure described by Oasa *et al.*[16] is applied. Briefly, the mole fraction of OLIG2-eGFP monomers (F_{monomer}) and homodimers ($F_{\mathrm{homodimer}}$), the sum of which is equal to one ($F_{\mathrm{monomer}} + F_{\mathrm{homodimer}} = 1$), were calculated using equations (S22) and (S23):

$$F_{\mathrm{monomer}} = \frac{4 - 2CPSM_{\mathrm{n}}}{3 - CPSM_{\mathrm{n}}} \quad \text{(S22)}$$

$$F_{\mathrm{homodimer}} = \frac{CPSM_{\mathrm{n}} - 1}{3 - CPSM_{\mathrm{n}}} \quad \text{(S23)}$$

where OLIG2-eGFP molecular brightness is reflected by counts *per* second and molecule (CPSM) and:

$$CPSM_{\mathrm{n}} = \frac{CPSM_{\text{OLIG2-eGFP,nuc}}}{CPSM_{\text{eGFP, cell}}} \ . \qquad \text{(S24)}$$

The concentration of OLIG2-eGFP monomers (c_{monomer}) and homodimers ($c_{\mathrm{homodimer}}$) in the cell nucleus is the product of the corresponding mole fraction and the total concentration as measured by FCS ($c_{\mathrm{monomer}} = F_{\mathrm{monomer}} \cdot c_{tot}$ and $c_{\mathrm{homodimer}} = F_{\mathrm{homodimer}} \cdot c_{tot}$), and the apparent dissociation constant of OLIG2-eGFP homodimers was determined as:

$$K_{\mathrm{d,app}}^{\text{(OLIG2-eGFP)2}} = \frac{c_{\mathrm{monomer}}^2}{c_{\mathrm{homodimer}}} \ . \qquad \text{(S25)}$$

The results of this analysis are summarized in Table S1.

Table S1. OLIG2-eGFP brightness analysis and determination of the apparent average dissociation constant of OLIG2-eGFP homodimers.

	Cytoplasm		Nucleus	
	Untreated	Treated	Untreated	Treated
CPSM / kHz	1.0 ± 0.7	1.0 ± 0.5	1.4 ± 0.7	1.1 ± 0.3
$\mathrm{CPSM_n}$	1.0	1.0	1.4	1.1
$\mathrm{F_{monomer}}$	1	1	0.77	0.94
$\mathrm{F_{homodimer}}$	0	0	0.23	0.06
c_{tot} / nM	50 ± 20	50 ± 40	210 ± 80	210 ± 130
c_{monomer} / nM	50 ± 20	50 ± 40	160	197
$c_{\mathrm{homodimer}}$ / nM	0	0	47	13
$K_{\mathrm{d,apparent}}^{\text{(OLIG2-eGFP)2}}$ / nM	*	*	560	3000

CPSM: Average OLIG2-eGFP molecular brightness determined by mpFCS. The bright pixels with CPSM > 2 were not included in this analysis.

$\mathrm{CPSM_n}$: Molecular brightness of OLIG2-eGFP against average molecular brightness of eGFP in live cell (Fig. 4, main text).

F: Mole fraction

c: Concentration

$K_{\mathrm{d,apparent}}^{\text{(OLIG2-eGFP)2}}$: Apparent dissociation constant of OLIG2-eGFP homodimers

S4. DETERMINING THE APPARENT DISSOCIATION CONSTANT FOR OLIG2-EGFP BINDING TO CHROMATIN DNA BY FCS. By fitting the theoretically derived equation for free 3D diffusion of two components to the ACCs recorded in the cell nucleus, (Fig. 5C2, Fig.S18B2 and C2) the mole fraction of free, $f_{\mathrm{free}}^{\mathrm{nuc}}$ and DNA-bound, $f_{\mathrm{bound}}^{\mathrm{nuc}}$, OLIG2-eGFP were determined.

The concentration of free, $[\text{OLIG2-eGFP}]_{\mathrm{free}}$, and bound, $[\text{OLIG2-eGFP}]_{\text{DNA-bound}}$, is the product of the corresponding mole fraction and the total OLIG2-eFFP concentration as measured by FCS, and the average apparent dissociation constant for OLIG2-eGFP binding to chromatin DNA was determined as:

$$K_{\mathrm{d,apparent}}^{\text{OLIG2-DNA}} = \frac{[\text{OLIG2-eGFP}]_{\mathrm{free}}^2}{[\text{OLIG2-eGFP}]_{\text{DNA-bound}}} \ . \qquad \text{(S26)}$$

To this aim, linear-regression analysis of $[\text{OLIG2-eGFP}]_{\mathrm{free}}^2 = f\,([\text{OLIG2-eGFP}]_{\text{DNA-bound}})$ was used to assess $K_{\mathrm{d,apparent}}^{\text{OLIG2-DNA}}$ from the slope (Fig. S19C1). Gaussian curve fitting to the histograms of apparent dissociation constant distribution is shown for measurements in the same cell before (Fig. S19C2) and after treatment (Fig. S19C3).

To examine the possible effects of slow processes and/or photobleaching on assessing the dissociation constants, we note that for processes with characteristic times that are very long, *i.e.* comparable with the signal acquisition time length, an apparently abrupt decay in the amplitude of the ACC is observed that arises because a small number of data points is collected at such long time scales (Fig. S5C2). While longer measurements would make it possible to collect more data points at long time scales, they would also increase the risk of photobleaching. Hence, there is a tradeoff between optimization of photobleaching and the signal acquisition length. To assess whether the ACCs could be fit satisfactorily with acceptable signal to noise using eq. (S1), $\alpha = 1$, $i = 2$, $T = 0$, we performed the chi-square test for goodness of fit. This analysis yielded chi-square (χ^2) values $\chi_{nuc}^2 = 2.7\times10^{-4}$ and $\chi_{cyt}^2 = 5.0\times10^{-5}$ in untreated OLIG2-eGFP expressing cells. For monomeric eGFP in live cells the goodness of fit using eq. (S1), $\alpha = 1$, $i = 1$, $T = 0$, yielded $\chi_{eGFP,cell}^2 = 3.2\times10^{-5}$ and for eGFP in buffer solution $\chi_{eGFP,sol}^2 = 2.4\times10^{-5}$. We therefore regard that ACCs reflecting OLIG2-eGFP diffusion and binding could be fit satisfactorily with acceptable signal to noise using eq. (S1), $\alpha = 1$, $i = 2$, $T = 0$.

In order to assess to what extent data points for which very long decay times were observed (such as data shown in Fig. S22, curves A-C) affect our analysis, we have compared the

relative fraction of bound OLIG2-eGFP in the cell nucleus ($f_{D,bound}^{nuc}$); the apparent diffusion behavior of OLIG2-eGFP in the cell nucleus affected by DNA binding, ($\tau_{D,bound}^{nuc}$) and the apparent dissociation constant of OLIG2-eGFP—DNA complexes ($K_{d,app}^{OLIG2-DNA}$) when including/excluding these data points.

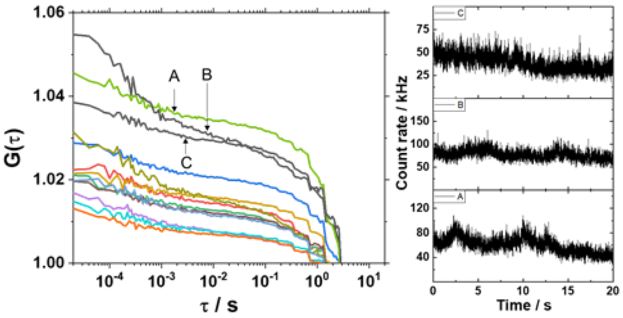

Figure S22 Fluorescence intensity fluctuation with very long characteristic times. Autocorrelation curves A-C are corresponding to fluorescence intensity fluctuation time series A-C in the right graph.

This analysis showed that while exclusion of data points with CPSM that are larger than twice the $CPSM_{eGFP}$, which are characterized by very long decay times (ACCs denoted A-C in Fig.S22) changes somewhat the actual values of the assessed variables: from $f_{D,bound}^{nuc}$ = (0.65 ± 0.10) to $f_{D,bound}^{nuc}$ = (0.60 ± 0.10); from $\tau_{D,bound}^{nuc}$ = (850 ± 500) ms to $\tau_{D,bound}^{nuc}$ = (600 ± 200) ms and from $K_{d,app}^{OLIG2-DNA}$ = (45 ± 30) nM to $K_{d,app}^{OLIG2-DNA}$= (60 ± 21) nM, the overall conclusions remain.

Moreover, we have tested whether adoption of different fitting models affects the interpretation of our data. In particular, the following models were tested: the two-component free diffusion model (eq. (S1), $\alpha = 1$, $T = 0$, $i = 2$; Fig. S23, red), the two-component anomalous diffusion model (eq. (S1), $\alpha \neq 1$, $T = 0$, $i = 2$; Fig. S23, green); the two-component diffusion model with anomalous first diffusion component and free second diffusion component (eq. (S1), $\alpha_1 \neq 1$, $\alpha_2 = 1$ $T = 0$, $i = 2$; Fig. S23, blue); the one-component free diffusion model with binding (eq. (S27)[17], $\alpha = 1$, $T = 0$, $i = 1$; Fig. S23, dark yellow) or the one-component anomalous diffusion model with binding (eq. (S27)[17], $\alpha \neq 1$, $T = 0$, $i = 1$; Fig. S23, dark blue). In eq. (S27)[17]:

$$G(\tau) = 1 + \frac{1}{N} \cdot \left[1 + \frac{T}{1-T} e^{-\frac{\tau}{\tau_T}}\right] \cdot \left(\frac{f_D}{\left(1 + \left(\frac{\tau}{\tau_{D_i}}\right)^{\alpha}\right) \sqrt{1 + \frac{w_{xy}^2}{w_z^2} \left(\frac{\tau}{\tau_{D_i}}\right)^{\alpha}}} + (1 - f_D) e^{-k_{off} \cdot \tau} \right) \quad \text{(S27)}$$

k_{off} denotes the dissociation rate constant, while all other parameters are the same as in eq. (S1).

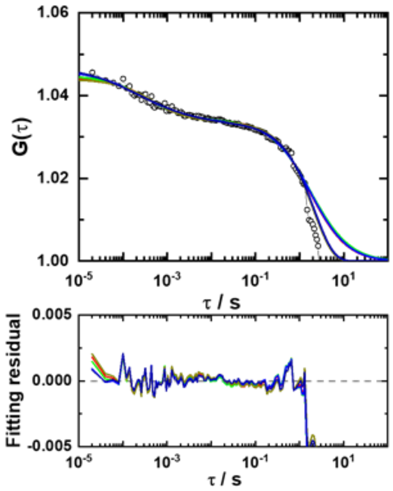

Figure S23: Analysis of OLIG2-eGFP diffusion and binding in the cell nucleus using different free and/or anomalous diffusion models with/without binding. ***Upper***: Single-pixel autocorrelation curve (open circles) fitted using the two-component free diffusion model (red), the two-component anomalous diffusion model (green), the two-component anomalous (first component) and free (second component) diffusion model (blue), the one-component free diffusion model with binding (dark yellow), or the one-component anomalous diffusion model with binding (dark blue). ***Bottom***: Corresponding fitting residuals.

As can be seen from the residuals analysis, all models can fit the ACC with similar chi-square values per degree of freedom: $\chi^2_{free,2c}$= 4.0 ×10^-6^, $\chi^2_{anom,2c}$ = 4.4 ×10^-6^, $\chi^2_{free+anom,2c}$= 4.1 ×10^-6^, $\chi^2_{free+binding,2c}$= 2.0 ×10^-6^ and $\chi^2_{free+binding,2c}$= 2.0 ×10^-6^. Moreover, the number of molecules (N), diffusion time of free OLIG2-eGFP (τ_{D1}) and the relative fraction of the second/binding component (f) are similar between all models: $N_{free,2c}$ = 22.6, $N_{anom,2c}$ = 22.3, $N_{free+anom,2c}$ = 21.6, $N_{free+binding,2c}$ = 22.7 and $N_{anom+binding,2c}$ = 21.2; $\tau_{D1}^{free,2c}$ = 390 µs, $\tau_{D1}^{anom,2c}$ = 340 µs, $\tau_{D1}^{free+anom,2c}$ = 270 µs, $\tau_{D1}^{free+binding,2c}$ = 450 µs, $\tau_{D1}^{anom+binding,2c}$ = 240 µs; $\tau_{D2}^{free,2c}$ = 1.7 s, $\tau_{D2}^{anom,2c}$ = 1.8 s, $\tau_{D2}^{free+anom,2c}$ = 1.8 s, $\tau_{Binding}^{free+binding,2c}$ = 2 s (k_{off} = 0.49 s^{-1}), $\tau_{Binding}^{anom+binding,2c}$ = 2.2 s (k_{off} = 0.46 s^{-1}); $f_2^{free,2c}$ = 0.77, $f_2^{anom,2c}$ = 0.76, $f_2^{free+anom,2c}$ = 0.73, $f_{binding}^{free+binding,2c}$ = 0.77 and $f_{binding}^{anom+binding,2c}$ = 0.71; for $\alpha^{free,2c}$ = 1, $\alpha^{anom,2c}$ = 0.95, $\alpha^{free+anom,2c}$ = 0.75, $\alpha^{free+binding,2c}$ = 1 and $\alpha^{anom+binding,2c}$ = 0.65. Interestingly, the anomalous diffusion exponent (α) was close to free diffusion ($\alpha \approx 1$) when the two-component anomalous diffusion model was applied, whereas the diffusion model with anomalous diffusion of the first component and free diffusion of the second component yielded α = 0.75, a value that is in-between values of α for eGFP monomer and tetramer.

Importantly, all diffusion models tested yielded congruent results and similar values for the apparent dissociation constant of OLIG2-eGFP–DNA-complexes, whereas a principal difference arises by adopting the diffusion and binding model (eq. (S27)), which yields the OLIG2-eGFP dissociation rate constant (k_{off}) instead of $\tau_{D,bound}^{nuc}$. According to this model, the mean life of the OLIG2-eGFP–DNA complex ($1/k_{off}$) is esti-

mated to be ≈ 2 s. While further studies are needed to establish which model correctly interprets the ACCs, it was previously shown that eGFP fluorescence is not changing upon eGFP-tagged transcription factor binding to DNA[18, 19], favoring our original interpretation.

For OLIG2-eGFP behavior in the cytoplasm, residuals- analysis showed that three different diffusion models: one-component anomalous diffusion (Fig. S24, green), two-component free diffusion model (Fig. S24, blue) and a two-component anomalous diffusion model (Fig. S24, red). could fit the ACC with similar chi-square values per degree of freedom: $\chi^2_{anom,1c}$= 2.8 ×10^{-5}, χ^2_{free} = 2.5 ×10^{-5} and $\chi^2_{anom,2c}$= 2.0 ×10^{-5}: yielding $N_{anom,1c}$ = 1.9, N_{free} = 5.5 and $N_{anom,2c}$ = 5.2; $\tau_D^{anom,1c}$ = 17 μs, τ_{D1}^{free} = 560 μs and τ_{D1}^{anom} = 350 μs; τ_{D2}^{free} = 70 ms and τ_{D2}^{anom} = 40 ms; f_2^{free} = 0.26 and f_2^{anom} = 0.26; for $\alpha^{anom,1c}$ = 0.34, α^{free} = 1 and $\alpha^{anom,2c}$ = 0.66.

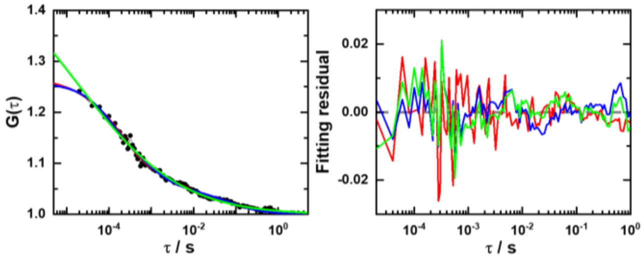

Figure S24. Two-component free *versus* anomalous diffusion model analysis of OLIG2-eGFP dynamics in the cytoplasm. ***Left***: Single-pixel autocorrelation curve (black symbols) fitted using a one-component anomalous diffusion model (green), two-component free diffusion model (blue) or a two-component anomalous diffusion model (red). ***Right***: Corresponding residuals.

However, the one-component anomalous diffusion model yields a very short diffusion time value, $\tau_D^{anom,1c}$ = 17 μs, that is inconsistent with protein diffusion. This model is therefore not applicable.

Finally, we show (Fig. S25) that similar results were obtained in different cells from three independently replicated experiments.

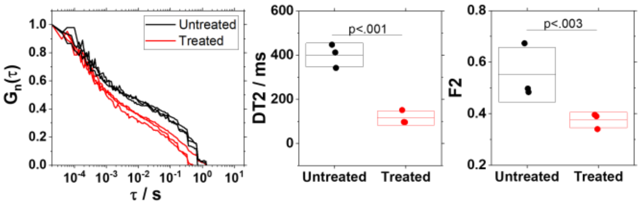

Figure S25: Effect of treatment with NSC 50467 on OLIG2-eGFP diffusion and binding in different cells. ***Left:*** Average autocorrelation curves (ACCs) reflecting OLIG2-eGFP diffusion and binding in the nucleus of untreated (black) and treated (red) cells. Measurements were taken in three independent experiments, starting from cell transfection, culturing, treatment and measurement. ***Middle:*** Diffusion time of bound OLIG2-eGFP (τ_{D2}) derived by fitting the ACCs shown to the left using a two-component free diffusion model. ***Right:*** Relative contribution of bound OLIG2-eGFP (f_2).

S5. STANDARD SOLUTIONS FOR mpFCS/FLIM CALIBRATION

Dilute aqueous suspension of 100 nm fluospheres (Fluo Spheres® Size Kit #2; Ex/Em: 505/515, Molecular Probes, Life Technologies Corporation, USA; D_{100} = 4.4 μm^2/s [2]) was used for the daily calibration of the mpFCS system. Occasionally, dilute aqueous suspension of quantum dots 525 (Qdot® 525 ITK™ Carboxyl Quantum Dots, Molecular Probes, Life Technologies Corporation, USA) and eGFP (purified by His-tag from E. coli) in 200 mM Phosphate buffer pH7.4, were used as secondary mpFCS calibration standards. For this purpose, the fluospheres and quantum dots were suspended in water and sonicated for 30 min before use. The standard solutions for mpFCS calibration were freshly prepared on the day of use and their concentration was measured by spFCS.

Single point Fluorescence Correlation Spectroscopy (spFCS) was performed using an uniquely modified ConfoCor3 system built on an LSM510 META platform, consisting of an inverted microscope for transmitted light and epifluorescence (Axiovert 200 M), the VIS-laser module comprising the Ar-ion (458, 477, 488 and 514 nm), HeNe 543 nm and the HeNe 633 nm lasers, the C-Apochromat 40×/1.2 N. A. W objective and silicon avalanche photodiodes (SPCM-AQR-1X; PerkinElmer) (Carl Zeiss MicroImaging GmbH, Jena, Germany)[20]. Dilute aqueous solution of ATTO488 (Sigma-Aldrich; $D_{ATTO488}$ = 400 μm^2/s [2]) was used for the daily calibration of the spFCS system. spFCS data were analyzed using the program for FCS data analysis that is part of the ConfoCor3 running software package.

For fluorescence lifetime (τ_f) measurements, the following standards were used: ATTO495 (Sigma-Aldrich: $\tau_{f,ATTO495}$ = 1.1 ns[21]), Rhodamine B (RhB; Sigma-Aldrich: $\tau_{f,RhB}$ = 1.56 ns[22]), enhanced Green Fluorescent Protein (eGFP: $\tau_{f,eGFP}$ = 2.4 ns and 2.6 ns[23, 24]), Rhodamine 6G (Rh6G; Sigma-Aldrich: $\tau_{f,Rh6G}$ = 3.79 ns[25]), ATTO488 (Sigma-Aldrich: $\tau_{f,ATTO488}$ = 4.2 ns and 4.16 ns[26, 27], BODIPY FL (Thermo Fisher Scientific: $\tau_{f,BODIPY}$ FL = 5.66 ns[28]), Rubrene (Sigma-Aldrich: $\tau_{f,Rubrene}$ = 7.4 ns[29, 30]). These substances were suspended in Dimethyl sulfoxide (DMSO) at a concentration of 10 μM, aliquoted and stored at -20 °C. For actual measurements, the fluorescence standards were thawed and, after reaching room temperature, diluted 100 - 1000 times using as a solvent: water for ATTO488, ATTO495, BODIPY FL, Rhodamine B and Rhodamine 6G; 200 mM Phosphate buffer pH7.4 for eGFP; and methanol for Rubrene.

For all measurements, in solution and in live cells, 8-well chambered cover glass (Nunc® Lab-Tek® II, Thermo Fisher Scientific) were used.

SUPPORTING REFERENCES

1. Krmpot, A. J.; Nikolić, S. N.; Oasa, S.; Papadopoulos, D. K.; Vitali, M.; Oura, M.; Mikuni, S.; Thyberg, P.; Tisa, S.; Kinjo, M.; Nilsson, L.; Terenius, L.; Rigler, R.; Vukojević, V., Functional Fluorescence Microscopy Imaging: Quantitative Scanning-Free Confocal Fluorescence Microscopy for the Characterization of Fast Dynamic Processes in Live Cells. Anal Chem 2019, 91 (17), 11129-11137.
2. Kapusta, P., Absolute Diffusion Coefficients: Compilation of Reference Data for FCS Calibration. Application Note (PicoQuant) Available at

https://www.picoquant.com/images/uploads/page/files/7353/appnote_diffusioncoefficients.pdf 2010.

3. Hess, S. T.; Webb, W. W., Focal volume optics and experimental artifacts in confocal fluorescence correlation spectroscopy. Biophys J 2002, 83 (4), 2300-17.
4. Vitali, M.; Bronzi, D.; Krmpot, A. J.; Nikolić, S. N.; Schmitt, F.; Junghans, C.; Tisa, S.; Friedrich, T.; Vukojević, V.; Terenius, L.; Zappa, F.; Rigler, R., A Single-Photon Avalanche Camera for Fluorescence Lifetime Imaging Microscopy and Correlation Spectroscopy. IEEE Journal of Selected Topics in Quantum Electronics 2014, 20 (6), 344-353.
5. Vallmitjana, A.; Dvornikov, A.; Torrado, B.; Jameson, D. M.; Ranjit, S.; Gratton, E., Resolution of 4 components in the same pixel in FLIM images using the phasor approach. Methods Appl Fluoresc 2020, 8 (3), 035001.
6. Digman, M. A.; Caiolfa, V. R.; Zamai, M.; Gratton, E., The phasor approach to fluorescence lifetime imaging analysis. Biophys J 2008, 94 (2), L14-L16.
7. Hanley, Q. S.; Clayton, A. H., AB-plot assisted determination of fluorophore mixtures in a fluorescence lifetime microscope using spectra or quenchers. J Microsc 2005, 218 (Pt 1), 62-7.
8. Clayton, A. H.; Hanley, Q. S.; Verveer, P. J., Graphical representation and multicomponent analysis of single-frequency fluorescence lifetime imaging microscopy data. J Microsc 2004, 213 (1), 1-5.
9. Weber, G., Resolution of the fluorescence lifetimes in a heterogeneous system by phase and modulation measurements. The Journal of Physical Chemistry 1981, 85 (8), 949-953.
10. Chen, S. J.; Sinsuebphon, N.; Rudkouskaya, A.; Barroso, M.; Intes, X.; Michalet, X., In vitro and in vivo phasor analysis of stoichiometry and pharmacokinetics using short-lifetime near-infrared dyes and time-gated imaging. J Biophotonics 2019, 12 (3), e201800185.
11. Fereidouni, F.; Esposito, A.; Blab, G. A.; Gerritsen, H. C., A modified phasor approach for analyzing time-gated fluorescence lifetime images. J Microsc 2011, 244 (3), 248-58.
12. Murakoshi, H.; Shibata, A. C. E., ShadowY: a dark yellow fluorescent protein for FLIM-based FRET measurement. Sci Rep 2017, 7 (1), 6791.
13. Lambert, T. J., FPbase: a community-editable fluorescent protein database. Nat Methods 2019, 16 (4), 277-278.
14. Oasa, S.; Vukojević, V.; Rigler, R.; Tsigelny, I. F.; Changeux, J. P.; Terenius, L., A strategy for designing allosteric modulators of transcription factor dimerization. Proc Natl Acad Sci U S A 2020, 117 (5), 2683-2686.
15. Tabu, K.; Ohnishi, A.; Sunden, Y.; Suzuki, T.; Tsuda, M.; Tanaka, S.; Sakai, T.; Nagashima, K.; Sawa, H., A novel function of OLIG2 to suppress human glial tumor cell growth via p27Kip1 transactivation. J Cell Sci 2006, 119 (Pt 7), 1433-41.
16. Oasa, S.; Mikuni, S.; Yamamoto, J.; Kurosaki, T.; Yamashita, D.; Kinjo, M., Relationship Between Homodimeric Glucocorticoid Receptor and Transcriptional Regulation Assessed via an In Vitro Fluorescence Correlation Spectroscopy-Microwell System. Sci Rep 2018, 8 (1), 7488.
17. Zhao, Z. W.; White, M. D.; Alvarez, Y. D.; Zenker, J.; Bissiere, S.; Plachta, N., Quantifying transcription factor–DNA binding in single cells in vivo with photoactivatable fluorescence correlation spectroscopy. Nature Protocols 2017, 12 (7), 1458-1471.
18. Vukojević, V.; Papadopoulos, D. K.; Terenius, L.; Gehring, W. J.; Rigler, R., Quantitative study of synthetic Hox transcription factor-DNA interactions in live cells. Proc Natl Acad Sci U S A 2010, 107 (9), 4093-8.
19. Papadopoulos, D. K.; Skouloudaki, K.; Engstrom, Y.; Terenius, L.; Rigler, R.; Zechner, C.; Vukojević, V.; Tomancak, P., Control of Hox transcription factor concentration and cell-to-cell variability by an auto-regulatory switch. Development 2019, 146 (12).
20. Vukojević, V.; Heidkamp, M.; Ming, Y.; Johansson, B.; Terenius, L.; Rigler, R., Quantitative single-molecule imaging by confocal laser scanning microscopy. Proc Natl Acad Sci U S A 2008, 105 (47), 18176-81.
21. Karedla, N.; Enderlein, J.; Gregor, I.; Chizhik, A. I., Absolute Photoluminescence Quantum Yield Measurement in a Complex Nanoscopic System with Multiple Overlapping States. J Phys Chem Lett 2014, 5 (7), 1198-202.
22. Mercadé-Prieto, R.; Rodriguez-Rivera, L.; Chen, X. D., Fluorescence lifetime of Rhodamine B in aqueous solutions of polysaccharides and proteins as a function of viscosity and temperature. Photochem Photobiol Sci 2017, 16 (11), 1727-1734.
23. Pepperkok, R.; Squire, A.; Geley, S.; Bastiaens, P. I., Simultaneous detection of multiple green fluorescent proteins in live cells by fluorescence lifetime imaging microscopy. Curr Biol 1999, 9 (5), 269-72.
24. Sarkisyan, K. S.; Goryashchenko, A. S.; Lidsky, P. V.; Gorbachev, D. A.; Bozhanova, N. G.; Gorokhovatsky, A. Y.; Pereverzeva, A. R.; Ryumina, A. P.; Zherdeva, V. V.; Savitsky, A. P.; Solntsev, K. M.; Bommarius, A. S.; Sharonov, G. V.; Lindquist, J. R.; Drobizhev, M.; Hughes, T. E.; Rebane, A.; Lukyanov, K. A.; Mishin, A. S., Green fluorescent protein with anionic tryptophan-based chromophore and long fluorescence lifetime. Biophys J 2015, 109 (2), 380-9.
25. Zander, C.; Sauer, M.; Drexhage, K. H.; Ko, D. S.; Schulz, A.; Wolfrum, J.; Brand, L.; Eggeling, C.; Seidel, C. A. M., Detection and characterization of single molecules in aqueous solution. Applied Physics B 1996, 63 (5), 517-523.
26. Urban, N. T.; Foreman, M. R.; Hell, S. W.; Sivan, Y., Nanoparticle-Assisted STED Nanoscopy with Gold Nanospheres. ACS Photonics 2018, 5 (7), 2574-2583.
27. Niehörster, T.; Löschberger, A.; Gregor, I.; Krämer, B.; Rahn, H. J.; Patting, M.; Koberling, F.; Enderlein, J.; Sauer, M., Multi-target spectrally resolved fluorescence lifetime imaging microscopy. Nat Methods 2016, 13 (3), 257-62.
28. Terpetschnig, E.; Jameson, D. M., Fluorescence Lifetime. ISS Technical note Available at http://www.iss.com/resources/pdf/technotes/Fluorescence_Lifetime.pdf 2005.
29. Boens, N.; Qin, W.; Basaric, N.; Hofkens, J.; Ameloot, M.; Pouget, J.; Lefevre, J. P.; Valeur, B.; Gratton, E.; vandeVen, M.; Silva, N. D., Jr.; Engelborghs, Y.; Willaert, K.; Sillen, A.; Rumbles, G.; Phillips, D.; Visser, A. J.; van Hoek, A.; Lakowicz, J. R.; Malak, H.; Gryczynski, I.; Szabo, A. G.; Krajcarski, D. T.; Tamai, N.; Miura, A., Fluorescence lifetime standards for time and frequency domain fluorescence spectroscopy. Anal Chem 2007, 79 (5), 2137-49.
30. Desportes, S.; Yatabe, Z.; Baumlin, S.; Génot, V.; Lefèvre, J.-P.; Ushiki, H.; Delaire, J. A.; Pansu, R. B., Fluorescence lifetime imaging microscopy for in situ observation of the nanocrystallization of rubrene in a microfluidic set-up. Chemical Physics Letters 2007, 446 (1), 212-216.
